# Machine Learning Early Detection of SARS‐CoV‐2 High‐Risk Variants

**DOI:** 10.1002/advs.202405058

**Published:** 2024-10-14

**Authors:** Lun Li, Cuiping Li, Na Li, Dong Zou, Wenming Zhao, Hong Luo, Yongbiao Xue, Zhang Zhang, Yiming Bao, Shuhui Song

**Affiliations:** ^1^ China National Center for Bioinformation Beijing 100101 China; ^2^ National Genomics Data Center Beijing Institute of Genomics Chinese Academy of Sciences Beijing 100101 China; ^3^ CAS Key Laboratory of Genome Sciences and Information Beijing Institute of Genomics Chinese Academy of Sciences Beijing 100101 China; ^4^ University of Chinese Academy of Sciences Beijing 100049 China

**Keywords:** haplotype network, high‐risk variant, machine learning, pre‐warning, SARS‐CoV‐2

## Abstract

The severe acute respiratory syndrome coronavirus 2 (SARS‐CoV‐2) has evolved many high‐risk variants, resulting in repeated COVID‐19 waves over the past years. Therefore, accurate early warning of high‐risk variants is vital for epidemic prevention and control. However, detecting high‐risk variants through experimental and epidemiological research is time‐consuming and often lags behind the emergence and spread of these variants. In this study, HiRisk‐Detector a machine learning algorithm based on haplotype network, is developed for computationally early detecting high‐risk SARS‐CoV‐2 variants. Leveraging over 7.6 million high‐quality and complete SARS‐CoV‐2 genomes and metadata, the effectiveness, robustness, and generalizability of HiRisk‐Detector are validated. First, HiRisk‐Detector is evaluated on actual empirical data, successfully detecting all 13 high‐risk variants, preceding World Health Organization announcements by 27 days on average. Second, its robustness is tested by reducing sequencing intensity to one‐fourth, noting only a minimal delay of 3.8 days, demonstrating its effectiveness. Third, HiRisk‐Detector is applied to detect risks among SARS‐CoV‐2 Omicron variant sub‐lineages, confirming its broad applicability and high ROC‐AUC and PR‐AUC performance. Overall, HiRisk‐Detector features powerful capacity for early detection of high‐risk variants, bearing great utility for any public emergency caused by infectious diseases or viruses.

## Graphical abstract

This study first validates a correlation between haplotype network features and the risk levels of SARS‐CoV‐2 variants. Building on this, HiRisk‐Detector, a machine learning algorithm, is developed for the early detection of high‐risk variants. The effectiveness, robustness, and generalizability of HiRisk‐Detector are confirmed using over 7.6 million SARS‐CoV‐2 genomes.

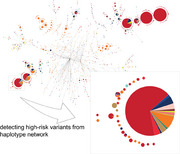

## 1 Introduction

The COVID‐19 pandemic was characterized by recurrent waves of cases, driven by the emergence of multiple high‐risk SARS‐CoV‐2 variants with increased capacity for transmission and evasion of existing immunity by infection or vaccine.^[^ ^1^ ^]^ Therefore, rapid and early detection of such high‐risk variants is critical to guide outbreak response.

To effectively address this challenge, the global health community has developed several surveillance systems to identify and monitor these variants. Currently, there are several methods for monitoring potential high‐risk variants of SARS‐CoV‐2, which are primarily based on early warning systems established by global public health organizations,^[^ ^2^, ^3^ ^]^ such as the World Health Organization (WHO), the United States Centers for Disease Control and Prevention, and the European Centers for Disease Control and Prevention. Among them, one representative method is that WHO classifies the potential high‐risk variants of SARS‐CoV‐2 into three categories—variants of concern (VOC), variants of interest (VOI), and variants under monitoring (VUM), with high risk to low risk according to transmissibility, virulence, immune escape, etc. Although this pre‐warning system is highly recognized and widely used, it requires the involvement of the WHO's Technical Advisory Group on SARS‐CoV‐2 Virus Evolution (TAG‐VE) to divide the risk of different variants. In other words, existing methods require extensive human knowledge and intervention and cannot fully automate the risk assessment. Critically, they have a certain degree of pre‐alarm lag, e.g., approximately one month by the WHO method.

On the contrary, several computational methods have been proposed in the past years to predict and analyze these variants based on a variety of molecular features primarily derived from genomes and metadata. These methods are generally classified into two types based on the need for reference sequence: reference‐free^[^ ^4^, ^5^ ^]^ and reference‐based.^[^ ^6^, ^7^, ^8^, ^9^ ^]^ Representative reference‐free methods include *k*‐mer‐based algorithm^[^ ^4^ ^]^ and dinucleotide composition representation (DCR) based algorithm.^[^ ^5^ ^]^ Specifically, the *k*‐mer‐based algorithm represents each SARS‐CoV‐2 amino acid sequence as *k*‐mer counts, and utilizes One‐class Support Vector Machines to identify VOC/VOI from a variety of variants.^[^ ^4^ ^]^ In contrast, the DCR algorithm involves DCR to parse the general human adaptation of RNA viruses and applies a three‐dimensional convolutional neural network to predict the adaptation of SARS‐CoV‐2 variants.^[^ ^5^ ^]^ While reference‐free methods offer the flexibility to study unknown genomes without the need for a reference sequence, they face challenges such as a potentially lower resolution in distinguishing highly similar sequences within the same species in the absence of a reference.

Regarding reference‐based methods, they utilize the Wuhan wild‐type variant as the reference sequence, as typified by VarEPS,^[^ ^6^ ^]^ spreading mutations‐based algorithm,^[^ ^7^ ^]^ and PyR_0_.^[^ ^8^ ^]^ Specifically, VarEPS focuses on analyzes the effects of mutations from the perspectives of genomics and structural biology, and uses these effects to predict the risk of SARS‐CoV‐2 variants based on random forest models. In comparison, the spreading‐mutations‐based algorithm develops a pipeline that predicts which individual amino acid mutations in SARS‐CoV‐2 are likely to become more prevalent in the coming months using logistic regression. By contrast, PyR0 provides a genome‐wide, automated approach for detecting viral lineages with increased fitness using Bayesian model. These methods have extensively examined the significance of features such as mutation frequency, spatial and temporal distribution, and protein structure within risk warning systems; nevertheless, they did not sufficiently make full use of evolutionary information in early warning of high‐risk variants.

Studying the evolutionary history of SARS‐CoV‐2 is crucial for the prevention and control of the outbreak. Evidence has shown that tracking the evolutionary history holds potential value in predicting successful mutations in future variants.^[^ ^10^ ^]^ Haplotype network is a common way to illustrate evolutionary history, offering valuable insights not only into the evolutionary relationships among sequences^[^ ^11^ ^]^ but also into the distinct transmission characteristics of various sequences.^[^ ^12^, ^13^ ^]^ Network features in the field of graph theory, such as node degree, betweenness, and network density, have been demonstrated to be valuable for studying transmission dynamics.^[^ ^14^ ^]^ Therefore, understanding the role of haplotype network features, and how they are related to virus transmissibility is crucial for performing risk assessment of SARS‐CoV‐2.

In this study, we initially investigated the potential of haplotype network features to aid in the early detection of SARS‐CoV‐2 high‐risk variants. Subsequently, we developed a haplotype network‐based machine learning algorithm, called HiRisk‐Detector. Building on this, we established a web‐based platform that utilizes the HiRisk‐Detector algorithm to detect high‐risk variants weekly in RCoV19 (https://ngdc.cncb.ac.cn/ncov/monitoring/risk)^[^ ^15^ ^]^ Based on empirical real data, we show that haplotype networks have great potential for detecting high‐risk variants and that HiRisk‐Detector is capable of timely and accurate detection of SARS‐CoV‐2 potential high‐risk variants with better robustness and generality, demonstrating its great potential for better improving health preparedness against public outbreak caused by any infectious disease.

## 2 Results

### 2.1 Correlation Between Haplotype Network Features and the Risk Levels of SARS‐CoV‐2 Variants

**Figure 1:**
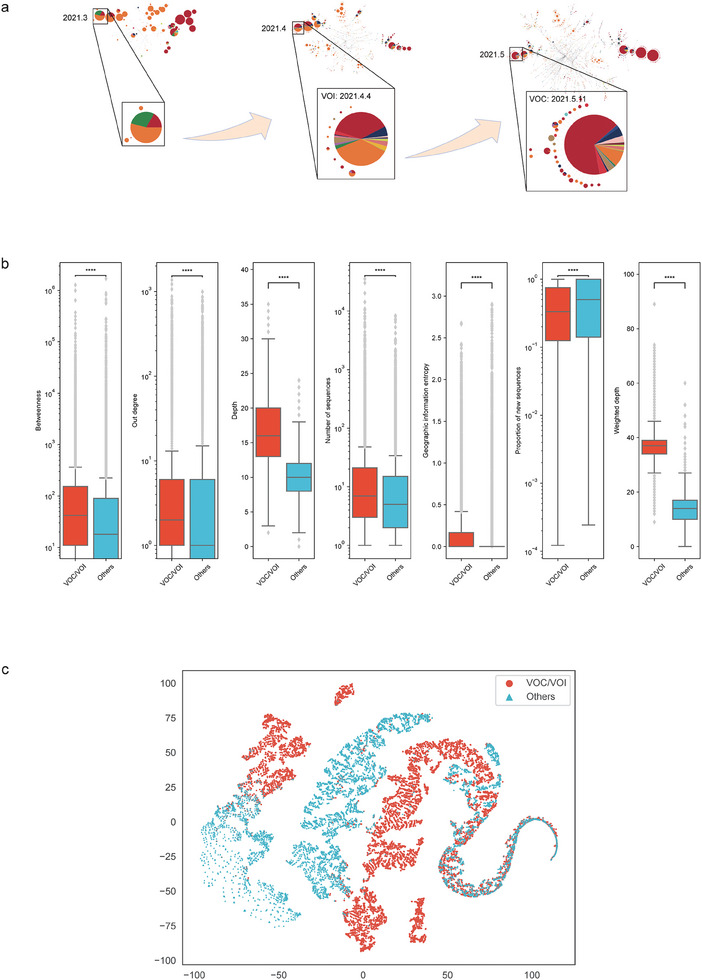
Haplotype network features of SARS‐CoV‐2 variants. a) A schematic diagram of haplotype network evolution, with obvious changes for some network features, e.g., node size and geographical transmission range. b) Box plot for the distribution of the features between VOC/VOI and others. The *p*‐values of the Mann‐Whitney test between VOC/VOI and others are labeled as ns: 5e‐2 < *p* ≤ 1, *: 1e‐2 < *p* ≤ 5e‐2, **: 1e‐3 < *p* ≤ 1e‐2, ***: 1e‐4 < *p* ≤ 1e‐3, ****: *p* ≤ 1e‐4. c) Haplotypes illustration in two‐dimensional space by t‐SNE (setting perplexity as 50, iteration as 1000, and random state as 42).

Haplotype networks have been demonstrated to be invaluable for tracing the transmission and evolution of SARS‐CoV‐2.^[^ ^16^ ^]^ By representing different haplotypes as nodes in the network and their connections as transmission and evolutionary relationships, we can gain insights into the virus's spread and genetic changes. A notable example is the analysis of publicly available genome sequences of SARS‐CoV‐2 from March to May 2021 globally. The resulting haplotype networks for the Delta variant exhibited unique characteristics, such as a rapidly increasing out‐degree of Delta haplotypes and an increase in the number of countries associated with them (**Figure** **1**a). This suggests that features within the haplotype network may aid in identifying high‐risk variants of SARS‐CoV‐2 that pose a threat to public health. Overall, the use of haplotype networks provides a powerful approach to understanding the complex dynamics of SARS‐CoV‐2 transmission and evolution. With the increasing availability of genomic data, it has become feasible to detect high‐risk variants of SARS‐CoV‐2 by examining the network characteristics of haplotypes.

To verify the hypothesis that there is a correlation between haplotype network features and the risk levels of SARS‐CoV‐2 variants, we constructed a series of haplotype networks from the *standard dataset* (Data S1, Supporting Information) at time points with a 7‐day interval ranging from September 5, 2020, to February 26, 2022 (see details of networks in Data S2, Supporting Information). We extracted seven features, including betweenness, out‐degree, depth, number of sequences, geographic information entropy, proportion of new sequences, and weighted depth (see definitions of these features in Supplementary Note S1, Supporting Information), from each haplotype, which has been observed within 7 days (called active haplotype, defined in Definition 3). To demonstrate the potential of haplotype networks for high‐risk variant detection, rather than the special mutations, these seven features were calculated only from haplotype networks without knowing the mutations of each haplotype.

By comparing the distributions of these network features between VOC/VOI and other variants, we calculated *p*‐values for each feature to assess the statistical significance of differences between high‐ and low‐risk haplotypes (Table S1, Supporting Information). Figure 1b shows that all *p*‐values are less than or equal to 1e‐4, which strongly implies that there are significant differences between the features of high‐ and low‐risk haplotypes. To visualize the haplotypes with those network features, we generated a random sample of 20 000 active haplotypes and reduced the dimension of features from seven to two by t‐Distributed Stochastic Neighbor Embedding^[^ ^17^ ^]^ (t‐SNE, setting perplexity as 50, iteration as 1000, and random state as 42) implemented in scikit‐learn.^[^ ^18^ ^]^ The 2D projection of haplotypes reveals clear boundaries between VOC/VOI and other variants for approximately three‐quarters of samples (Figure 1c). Together, our results suggest that these haplotype network features have the potential to help detect high‐risk variants of SARS‐CoV‐2.

### 2.2 Detection of SARS‐CoV‐2 High‐Risk Variants Based on Haplotype Network Features and Machine Learning Methods

To validate the actual ability of these haplotype network features in identifying high‐risk variants of SARS‐CoV‐2, we employed them along with machine learning methods for retrospective analysis and verification on historical high‐risk variants of SARS‐CoV‐2. We used k‐fold cross‐validation to select the optimal features and machine learning model, and evaluated their performance on the testing set.

#### 2.2.1 Dataset Preparation

We randomly partitioned all active haplotypes into a training set and a testing set, with the former containing 80% of the active haplotypes, and the latter containing the remaining 20%. To ensure robustness, we further randomly split the training set into five groups with the same number of haplotypes for k‐fold cross‐validation.

#### 2.2.2 Feature and Model Selection (k‐Fold Cross‐Validation)

We performed fivefold cross‐validation on the training set to select the optimal machine learning method and features. Specifically, we evaluated the cross‐validation scores for three state‐of‐the‐art machine learning methods, including LightGBM,^[^ ^19^ ^]^ random forest,^[^ ^20^ ^]^ and logistic regression,^[^ ^21^ ^]^ along with each combination of the seven haplotype network features mentioned above. Due to the high transmissibility of high‐risk variants, the high‐risk haplotypes account for ≈ 80.9% (= 310 299 / 383 691) of the total number of haplotypes, after removing duplicates. To reduce the impact of data imbalance, we adopted cost‐sensitive learning^[^ ^22^ ^]^ by setting the parameter “class_weight” to “balanced” in scikit‐learn. Note that for each machine learning method, we utilized only its default parameters except for “class_weight”. Figure S1 (Supporting Information) shows the average cross‐validation score of each machine learning method (top 10 combinations of features), showing that the random forest method with five features, including betweenness, out‐degree, depth, geographic information entropy, and weighted depth, achieves the highest cross‐validation score. Therefore, we selected the random forest method and these five features as the hyperparameters for the model.

#### 2.2.3 Performance on Testing Data Set

**Figure 2:**
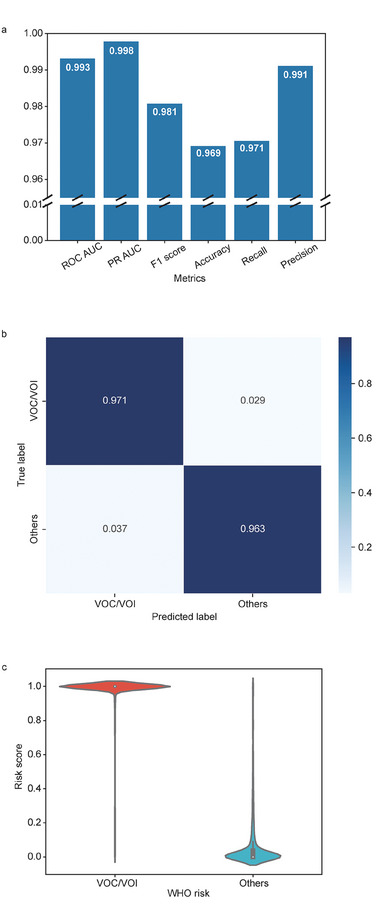
Performance on testing set. The model was retrained on the entire training set, with the selected AI method (random forest) and five features (betweenness, out‐degree, depth, geographic information entropy, and weighted depth); and tested on the testing set. a) shows six metrics including ROC‐AUC, PR‐AUC, F1 score, accuracy, recall, and precision on the testing set. The heights of the bars represent the values of each metric. b) The normalized confusion matrix. c) Risk score of VOC/VOI and the other variants. The risk score is the probability that a test sample is a high‐risk variant, which is predicted by the trained model.

To assess the final performance, we trained the model on the entire training set using the selected five features and the random forest method and then evaluated its performance on the testing set. Six metrics of performance were evaluated on the testing set, showing that all these metrics, including ROC‐AUC, PR‐AUC, F1 score, accuracy, recall, and precision, exceed 0.96 (**Figure** **2**a). The normalized confusion matrix (Figure 2b) shows that the true positive rate and true negative rate are both greater than 0.96, demonstrating a high level of accuracy in correctly identifying positive and negative instances. Furthermore, the false negative rate and false positive rate are both below 0.04, implying a low rate of incorrectly classifying instances. The violin plot of the risk score shows that the VOC/VOI has a median and average risk score of 1.00 and 0.97, respectively, indicating an excellent performance. In contrast, the other variants have significantly lower scores with median and average risk scores of 0.00 and 0.07, respectively (Figure 2c). This suggests that the model is highly effective in identifying VOC/VOI. To summarize, our study findings indicate that these five haplotype network features are powerful in helping detect high‐risk variants of SARS‐CoV‐2.

### 2.3 Performance with Label Noise and Data Incompleteness

There is often a lag between the emergence of a new high‐risk variant and its official designation as a VOC/VOI by organizations, such as WHO and CDC. For example, the average lag time between the designation dates of each existing VOC/VOI and their earliest submission dates is 29.5 days (Table S2, Supporting Information). As a result, in practical applications, it is difficult to assign accurate labels to high‐risk variants in a training set until WHO designs them as VOC/VOI. In addition, strains submitted after the detection time are not available at the time of detection. All these factors prompted us to evaluate the performance of data characterized by label noise and data incompleteness.

**Figure 3:**
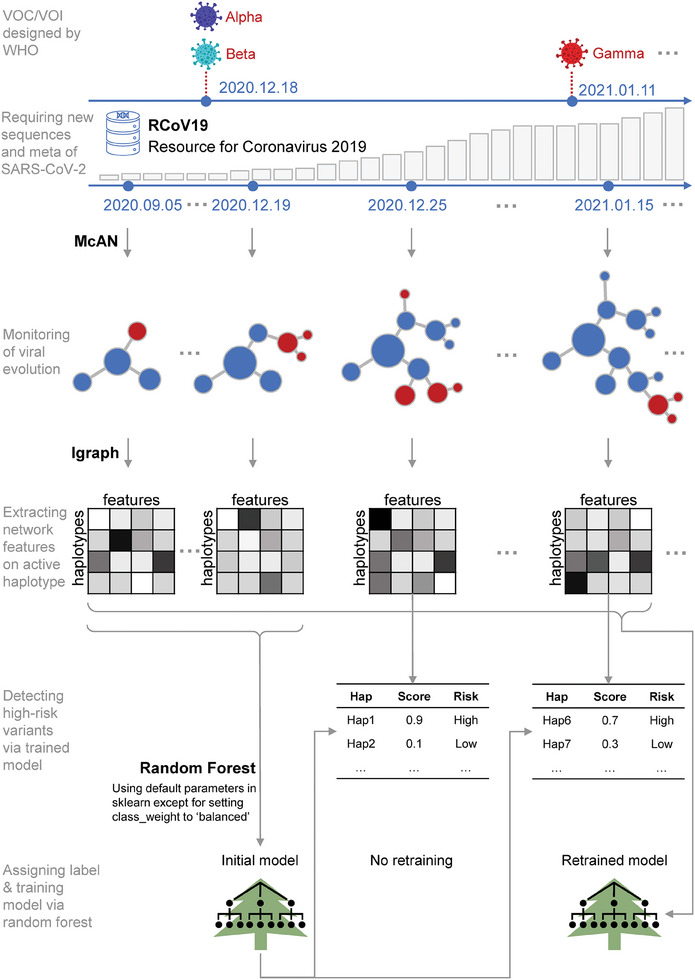
Schematic illustration for HiRisk‐Detector algorithm. The process began by establishing surveillance time points every 7 days starting from September 5, 2020, to monitor the evolution and risk of SARS‐CoV‐2. The initial phase involved training a model using active haplotypes from networks constructed between September 5, 2020, and December 19, 2020, shortly after the WHO designated the first VOC/VOI. Subsequently, for each time point after December 18, 2020, a haplotype network was formed from all available sequences, from which key features were extracted to predict risk scores for each haplotype. Haplotypes with scores over 0.5 were classified as high‐risk. Additionally, if the WHO identified new variants between two consecutive surveillance points, the model was retrained with data from all networks constructed up to the current time point.

**Figure 4:**
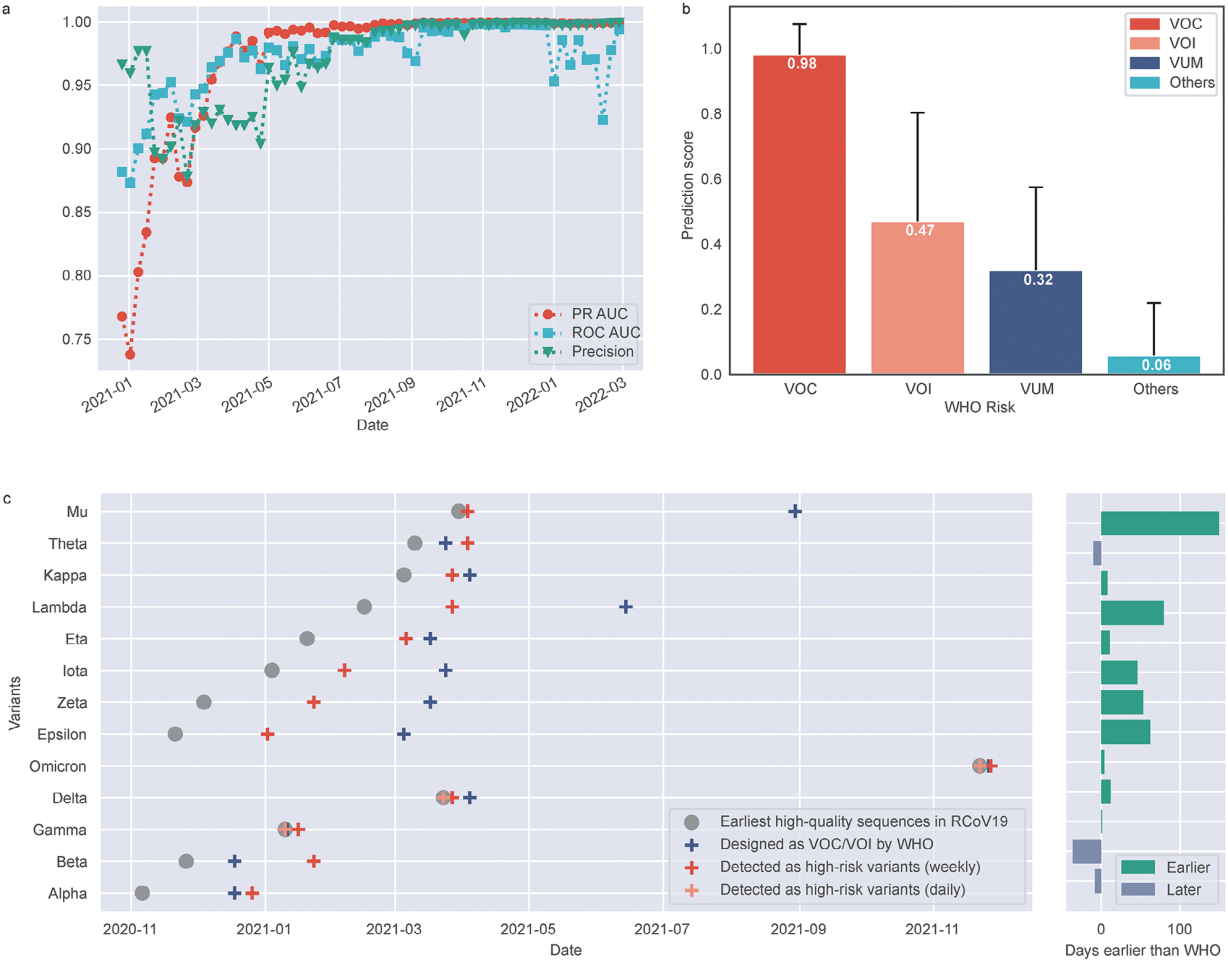
Results of retrospective detection with data incompleteness and label noise on the standard dataset. HiRisk‐Detector was tested on the *standard dataset* containing 4396290 strains with submission data no later than February 28, 2022, to evaluate performance under data incompleteness and label noise. a) PR‐AUC, ROC‐AUC, and precision on each week. b) HiRisk‐Detector of each WHO risk, including VOC, VOI, VUM, and the other variants. The bars' heights represent each WHO risk's average risk score. The error bars represent the standard deviation. c) shows the earliest date that a VOC/VOI was detected as a high‐risk variant by HiRisk‐Detector, the earliest date that the VOC/VOI was submitted to public databases, and the date that the VOC/VOI was designed as VOC/VOI by WHO. The differences between the earliest date that a VOC/VOI was detected as a high‐risk variant by HiRisk‐Detector and the date that the variant was designed as VOC/VOI by WHO are illustrated on the right side. If the former is earlier than the latter, the bar is colored by Persian green; otherwise, the bar is colored by wild blue yonder.

Therefore, we developed haplotype network‐based machine learning algorithm, HiRisk‐Detector (illustrated in **Figure** **3** and described in Experimental Section); and assessed its efficacy on the standard dataset. To evaluate the performance of HiRisk‐Detector, we calculated PR‐AUC, ROC‐AUC, and precision at each surveillance time point after December 18, 2020. **Figure** **4**a shows that all precision values are higher than 0.85 (Table S3, Supporting Information). In addition, we noted a steady increase in PR‐AUC, ROC‐AUC, and precision over time, ultimately approaching 1 due to the expansion of sample size and enhanced accuracy of labels in the training set. We calculated the average risk score of all active haplotypes within each WHO risk. The result shows that the average risk score of VOC is as high as 0.98, while other variants (the variants that are not VOC, VOI, or VUM) have a lower average score of 0.06 (Figure 4b; Figure S2, Supporting Information). Moreover, we observed that the average risk scores of VOC, VOI, VUM, and other variants decreased in turn. These results suggest that HiRisk‐Detector is robust to label noise and data incompleteness. This can be attributed to the inherent tolerance of random forests for outliers and noise, coupled with their reduced susceptibility to overfitting. Figure 4c shows that HiRisk‐Detector successfully identified all 13 VOC/VOI, with an average pre‐warning time of 27 days earlier than WHO's announcement. Furthermore, we calculated the number of high‐quality and complete sequences available for each VOC/VOI before HiRisk‐Detector detected at least one haplotype of this variant as a high‐risk variant (Figure S3, Supporting Information). Our findings indicate that to identify a VOC/VOI as a high‐risk variant, a minimum of 99.9 and 51.0 sequences of this variant are necessary, based on the mean and median values respectively. Taken together, these results indicate that HiRisk‐Detector is capable of delivering earlier warnings for high‐risk variants with high precision.

To enable the visualization of pre‐warning results and the traceability of historical data, we further developed a web‐based platform (https://ngdc.cncb.ac.cn/ncov/monitoring/risk)^[^ ^15^ ^]^ that uses the HiRisk‐Detector to update high‐risk variants in RCoV19 on a weekly basis.

### 2.4 Effect of Sequencing Intensity

Over the past year, the availability of new SARS‐CoV‐2 sequences has declined (Figure S4, Supporting Information), largely due to cost and resource limitations. Large‐scale viral sequencing demands significant resources and funding, and as the pandemic has subsided, many countries have reduced their investments in these activities. Consequently, this reduction in sequencing capacity has led us to assess the performance of HiRisk‐Detector in scenarios where sequencing resources are constrained.

**Figure 5:**
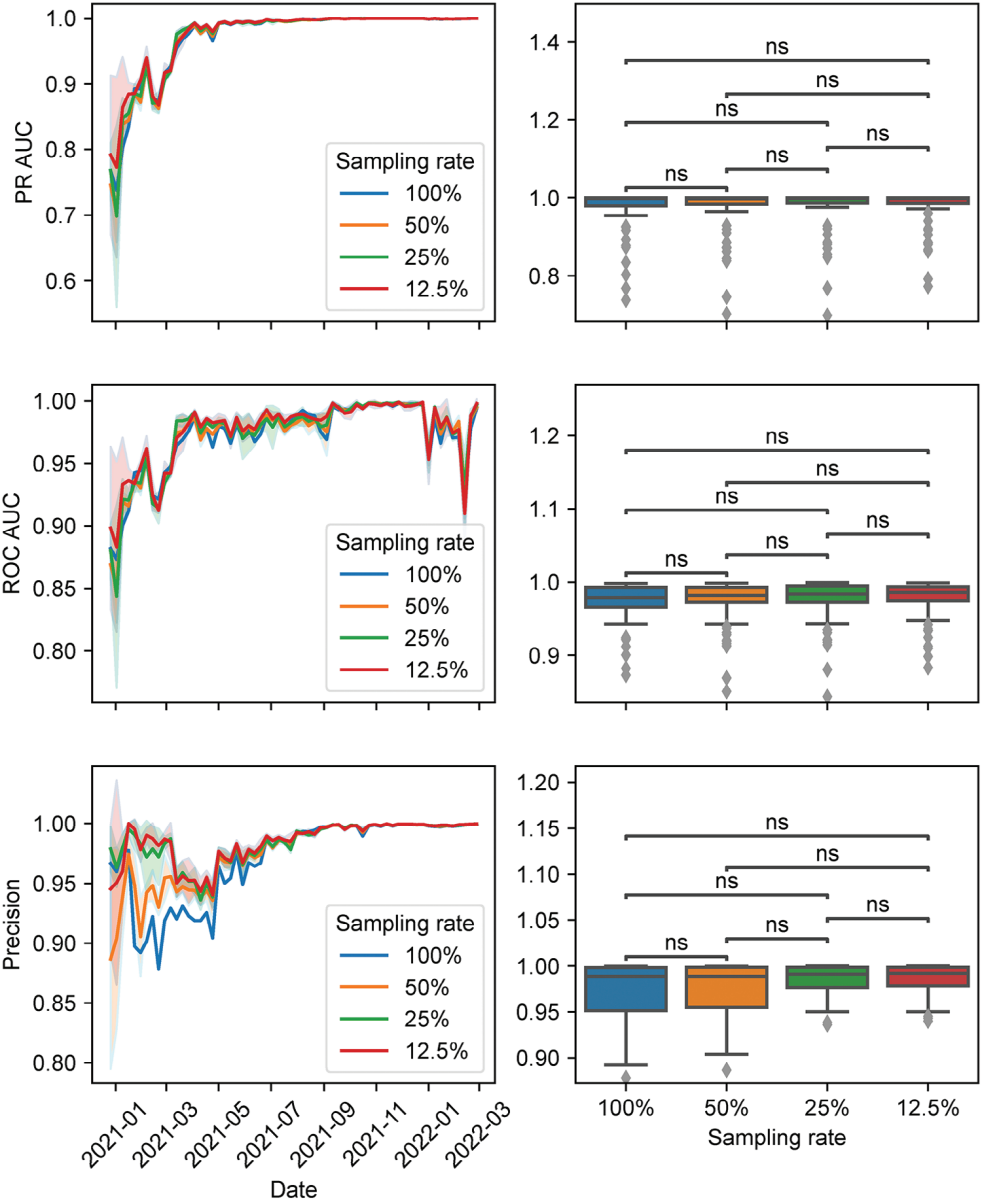
Performance at different sampling rates. The first column shows the variation of metrics, including PR‐AUC, ROC‐AUC, and precision, at each sampling rate, such as 50%, 25%, and 12.5%. The second column shows the boxplot of each metric at each sampling rate. The *p*‐values of the Mann‐Whitney test between each sampling rate are labeled as ns: 5e‐2 < *p* ≤ 1, *: 1e‐2 < *p* ≤ 5e‐2, **: 1e‐3 < *p* ≤ 1e‐2, ***: 1e‐4 < *p* ≤ 1e‐3, ****: *p* ≤ 1e‐4. The first, second, and third rows show the results of PR‐AUC, ROC‐AUC, and precision, respectively.

As sequencing intensity decreases, the number of training samples available at each detection time point diminishes, posing significant challenges to the machine learning model's performance due to the limited data available for effective learning. To assess the model's robustness, we simulated various levels of sequencing intensity reduction, mirroring real‐world scenarios where sequencing resources may be constrained. We conducted experiments on the *standard dataset* with an actual sequencing intensity of 1.01%. To evaluate the performance of the algorithm under reduced sequencing conditions, we downsampled the dataset to intensities of 0.51%, 0.25%, and 0.13%, corresponding to 50%, 25%, and 12.5% of the actual intensity, respectively. Subsequently, we applied the HiRisk‐Detector algorithm as outlined in the previous section to these downsampled datasets. To enhance the reliability of our findings, we repeated each experiment three times. **Figure** **5** shows that there is no significant change in performances including PR‐AUC, ROC‐AUC, and precision when the sampling rate is reduced to 50%, 25%, and 12.5%. Furthermore, as the sequencing intensity decreases from 1.01% to 0.25%, the average warning time is only delayed by 3.7 days (Figure S5, Table S4, Supporting Information), and precision at each time point remains above 0.85 (Figure 5). These results indicate that HiRisk‐Detector maintains high performance even at lower sequencing intensities, demonstrating its robustness to variations in sequencing intensity.

### 2.5 Generalization of HiRisk‐Detector for Omicron Sub‐Lineages

On November 24, 2021, scientists in Botswana and South Africa discovered the most potent SARS‐CoV‐2 variant, which caused global concern. On November 26, 2021, the WHO's TAG‐VE classified this new variant as a VOC and named it “Omicron”. Since its emergence, Omicron variants have continued to evolve genetically and antigenically, with an expanding range of sub‐lineages. There is consensus among experts in WHO's TAG‐VE that compared to previous variants, Omicron represents the most divergent VOC seen to date. The previous system classified all Omicron sub‐lineages as part of the Omicron VOC, which lacked the necessary granularity to compare new descendent lineages with altered phenotypes to the Omicron parent lineages. Therefore, in mid‐March 2023, WHO updated its tracking system and working definitions for VOC, VOI, and VUM of SARS‐CoV‐2 to better monitor the current variant landscape, which is dominated by Omicron descendent lineages (https://www.who.int/en/activities/tracking‐SARS‐CoV‐2‐variants). Correspondingly, we have also updated HiRisk‐Detector for risk assessment of Omicron sub‐lineages. We changed the dataset to the post‐Omicron dataset and assigned labels on the training set according to the WHO's new tracking system, while other steps remain the same in HiRisk‐Detector (see Experimental Section).

**Figure 6:**
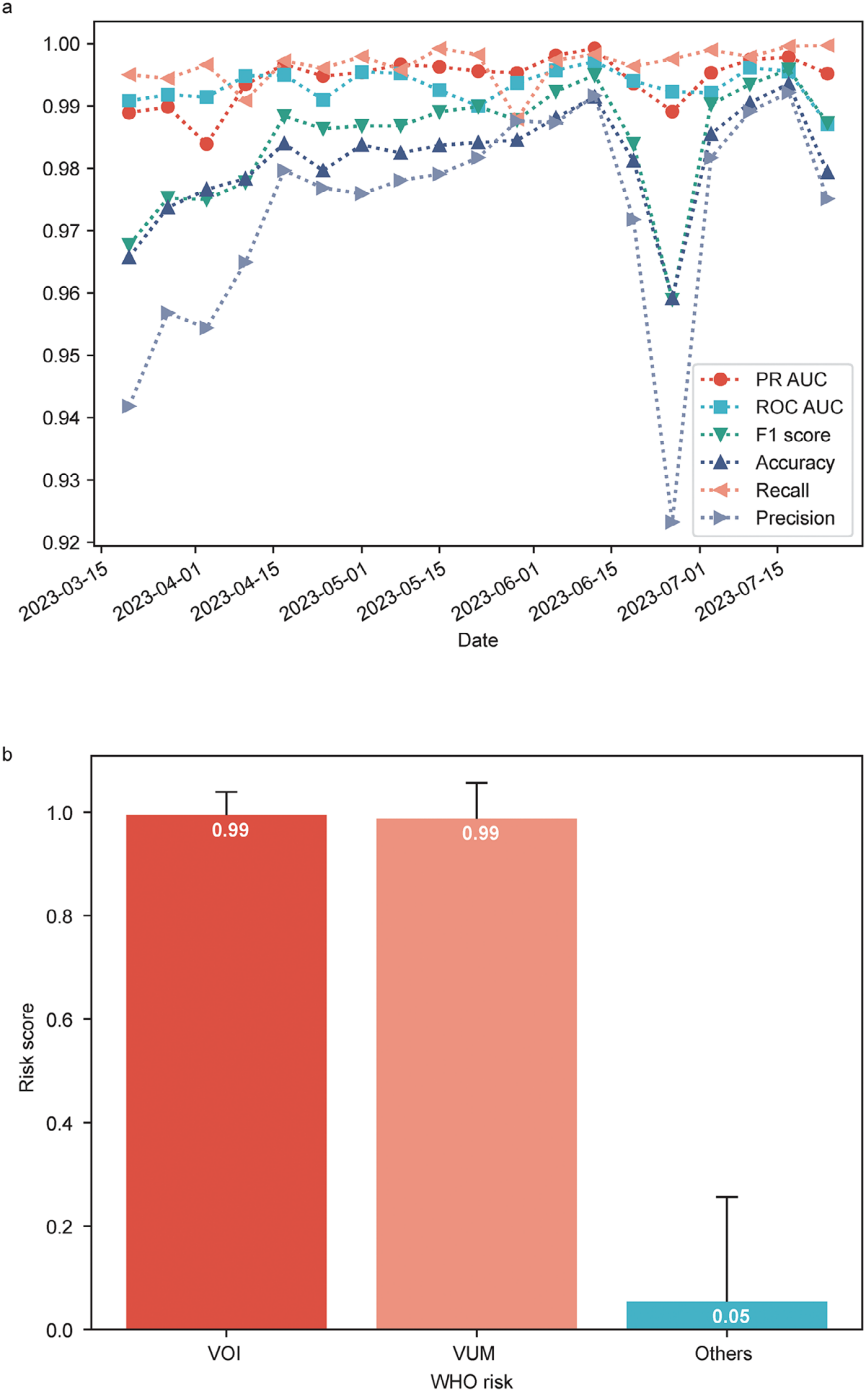
Results of retrospective detection with data incompleteness and label noise on the post‐Omicron dataset. a) PR‐AUC, ROC‐AUC, F1 score, accuracy, recall, and precision on each week. b) The risk score of each WHO risk, including VOI, VUM, and the other variants. The bars' heights represent each WHO risk's average risk score. The error bars represent the standard deviation.

The results show that metrics including PR‐AUC, ROC‐AUC, F1 score, accuracy, recall, and precision are all higher than 0.92 at all testing time points (**Figure** **6**a; Table S5, Supporting Information). The average risk score of VOI and VUM is as high as 0.99, while the average risk score of other variants is only 0.05 (Figure 6b). Additionally, the VOI/VUM (designed no earlier than March 20, 2023) was detected on average 29.8 days earlier than WHO's announcement (**Table** **1**).

**Table 1:** Key dates of each VOI/VUM from experiments on the post‐Omicron dataset.

PANGO lineage^a)^	Detection date^b)^, ^c)^	Designed as VOI/VUM^c)^, ^d)^	Earlier than WHO (days)^e)^	Earliest strains^c)^, ^f)^
XBB.2.3	2023/3/20	2023/5/17	58	2023/1/4
XBB.1.9.2	2023/3/20	2023/4/26	37	2023/1/31
XBB.1.16	2023/4/3	2023/4/17	14	2023/3/21
XBB.1.9.1	2023/3/20	2023/3/30	10	2022/12/13
CH.1.1^g)^	2023/3/20	2023/2/8	−40	2022/10/13
XBB.1.5^g)^	2023/3/20	2023/1/11	−68	2022/11/14
XBB^g)^	2023/3/20	2022/10/12	−159	2022/9/12
BA.2.75^g)^	2023/4/10	2022/7/6	−278	2022/6/21

^a)^ Include all VOI/VUM variants defined by WHO's new tracking system, as of July 2023;

^b)^ The date of a given variant detected as a high‐risk variant by HiRisk‐Detector;

^c)^ The date format is year/month/day;

^d)^ The date of a variant designed as VOI/VUM by WHO's new tracking system;

^e)^ The difference between the 3rd column and the 2nd column;

^f)^ The date of earliest high‐quality and complete strains based on the submission date extracted from metadata in RCoV19;

^g)^ Variants that were designed as VOI/VUM before March 2023.

## 3 Discussion and Conclusion

In this work, we explored the potential of haplotype network to help detect high‐risk variants of SARS‐CoV‐2 and developed a haplotype network‐based machine learning algorithm, HiRisk‐Detector. Subsequently, we established a web‐based platform to weekly update SARS‐CoV‐2 high‐risk variants, providing real‐time information to researchers and public health authorities. Our results indicate that haplotype network features hold great promise for detecting high‐risk variants. HiRisk‐Detector is capable of timely and accurate detection of high‐risk variants, exhibits robustness against label noise, incomplete data, and low sequencing intensity, and can be generalized to assess the risk of Omicron sub‐lineages.

Several previous studies have used mutations as inputs to machine learning models, which may lead to over‐reliance on some key mutations. To reduce the effect of mutations, we calculate seven features solely from the haplotype network without considering which specific mutations are present on each haplotype and edge. Figure S6 (Supporting Information) shows that the precision of the network‐based workflow is much higher than that of the k‐mer‐based workflow proposed in.^[^ ^4^ ^]^ Our proposed approach highlights the significant role of population genomic data in detecting high‐risk variants.

The seven features can be divided into two categories: node‐feature and edge‐feature, according to whether a feature is calculated from node or edge. The node‐feature contains three features including geographic information entropy, number of sequences, and proportion of new sequences. In contrast, the edge‐feature contains four aspects including betweenness, out‐degree, depth, and weighted depth. Our analysis revealed that only geographic information entropy is a node‐feature among the five selected features in HiRisk‐Detector, while all others are edge‐features. This suggests that evolutionary relationships play a crucial role in detecting high‐risk variants.

Sequencing intensity plays a crucial role in the early detection and warning of SARS‐CoV‐2 high‐risk variants. However, due to differences in sequencing capabilities and levels, sequencing intensity varies across countries and regions. Additionally, as people's attention toward the pandemic wanes in the post‐pandemic era, the sequencing intensity has decreased, which can result in delays in identifying high‐risk variants. Therefore, it is essential to develop tools that are not affected or minimally affected by changes in sequencing intensity. This will ensure that we continue to monitor and track the evolution of the virus effectively. To tackle the challenges posed by insufficient sampling of rare high‐risk variants resulting from low sequencing intensity, we integrated haplotype networks into the methodologies for detecting high‐risk variants. By incorporating these networks, the model is equipped to discern the underlying evolutionary and transmission dynamics common among high‐risk variants present in the haplotype network. This approach significantly mitigates the effects of incomplete rare high‐risk variant samples during the model training phase, enhancing the robustness and accuracy of the detection process.

The JN.1 variant has maintained its prominence as the predominant variant, as the initial paper completed. Our HiRisk‐Detector has consistently tracked JN.1, assigning it a sustained high‐risk score (>0.8) since November 7, 2023, as documented in the online monitoring resource (https://ngdc.cncb.ac.cn/ncov/monitoring/risk). This serves as yet another compelling illustration of the algorithm's capacity to effectively surveil high‐risk haplotypes.

HiRisk‐Detector has been demonstrated to be a valuable tool in detecting high‐risk variants, but it still has limitations in several aspects. First, acquiring labeled data is essential for the training or retraining models. This implies that HiRisk‐Detector process heavily relies on authoritative tracking systems such as WHO. Second, the time complexity of all known haplotype network construction algorithms, including TCS,^[^ ^23^ ^]^ MSN, MJN,^[^ ^24^ ^]^ and McAN,^[^ ^25^ ^]^ are not lower than O(*n* ^2^) in the worst‐case scenario, where *n* is the number of haplotypes. As a result, the risk detection method based on haplotype network features is relatively time‐consuming in the feature extraction stage. Therefore, future research should focus on reducing the computational time required for constructing haplotype networks. Third, in the current workflow, we have manually chosen seven candidate haplotype network features for training the model, relying on prior work experience and data analysis. However, this approach to feature extraction is somewhat arbitrary and lacks automatic capability. Consequently, employing automatic feature extraction methods such as Graph Convolutional Networks (GCN)^[^ ^26^ ^]^ could be advantageous in improving the performance of HiRisk‐Detector. Fourth, the presence of degenerate bases in the genome sequence may arise from mixed infections or intra‐host evolution. Currently, our algorithm focuses on consensus sequences, which may overlook these complexities. To improve the detection of such variations, we plan to refine our algorithm by incorporating a step that extracts non‐consensus sequences from raw next generation sequencing data. This enhancement will allow for risk monitoring at a more detailed and finer granularity.

In summary, it is a new perspective to identify high‐risk variants of SARS‐CoV‐2 using haplotype network features. The high accuracy and robustness of HiRisk‐Detector bear promise to improve public health preparedness against the ongoing evolving virus. HiRisk‐Detector has the potential to be generalized to monitor other rapidly evolving pathogens with sufficient genomic data.

## 4 Experimental Section

### Data Collection and Preparation

A total of 7 662 981 complete and high‐quality SARS‐CoV‐2 genome sequences (Supplementary Note 2, Supporting Information) along with their associated metadata were downloaded from RCoV19 (https://ngdc.cncb.ac.cn/ncov/).^[^ ^27^, ^28^ ^]^ This includes all available data as of July 27, 2023. To ensure accuracy, it has standardized the metadata and removed any sequences that were missing Pango lineage, submission date, or sampling location information. These data will no longer be used in subsequent analyses.

In order to be suitable for different application scenarios, two datasets were constructed for assessing HiRisk‐Detector. Since February 2022, the Omicron variants of SARS‐CoV‐2 have become the dominant strain, accounting for over 98% of publicly available sequences. Therefore, a dataset (called a *standard dataset*, Data S1, Supporting Information) was defined as all data collected before February 28, 2022, which includes a total of 4396290 SARS‐CoV‐2 sequences and associated metadata submitted before that date. In addition, to evaluate the performance of HiRisk‐Detector on Omicron sub‐lineages, another dataset (called *post‐Omicron dataset*, Data S3, Supporting Information) consisting of 4661821 SARS‐CoV‐2 sequences submitted between November 22, 2021, and July 27, 2023 was constructed. Notably, November 22, 2021, was the date of submission of the first Omicron variant sequence included in RCoV19.

In order to assign labels to haplotypes, the alias file of PANGO lineages was downloaded, which included 271 aliases (https://github.com/cov‐lineages/pango‐designation/alias_key.json).^[^ ^29^, ^30^, ^31^ ^]^ Subsequently, the association between PANGO lineage, WHO label, and WHO risk was documented based on the information provided on the official WHO website (https://www.who.int/en/activities/tracking‐SARS‐CoV‐2‐variants).

### Haplotype Network Construction

The sequence mutations were identified by comparing the retained sequences against the earliest released SARS‐CoV‐2 genome (GenBank: MN908947.3). To accurately characterize the diversity of haplotypes, degenerate mutations that did not belong to the [A/T/G/C] bases were eliminated. The population mutation frequency (PMF) was then calculated for each mutation within a one‐week time window. Mutations with PMF greater than 0.005 in non‐UTR regions were selected for haplotype network construction.

With the progression of the pandemic, a minority of existing variants were continuously spreading while new variants continued to emerge, leading to the constant evolution of the SARS‐CoV‐2 haplotype network over time. Haplotype network was any graph used to represent evolutionary relationships between a set of haplotypes.^[^ ^32^ ^]^ Note that, the mutations in each haplotype were not contained in haplotype network in this work (Definition 1). To track the evolution of SARS‐CoV‐2, a range of equally spaced time points were generated and a series of haplotype networks were constructed using McAN^[^ ^25^ ^]^ with default parameters based on the sequences available at these time points.

Definition 1 (Haplotype network) Haplotype network is defined as a graph *G*  = (*V*, *E*) , where $V=\left\{h_{i}|i=0,1,\;\text{…},m\right\}$ is a collection of haplotypes, *h_i_* is the (*i* + 1)*th* haplotype, *m* is the number of haplotypes in haplotype network *G*, *E*  = {*e_ij_* = (*h_i_*,*h_j_*)}  is the collection of edges, representing the evolutionary relationships among haplotypes. In graph *G*, each haplotype *h_i_* ∈ *V* has a node weight (the number of strains in the haplotype *h_i_*), denoted as *w_v_* (*h_i_*), and each edge *e_ij_* has an edge weight *w_e_* (*e_ij_*), defined as the number of differential nucleotide mutations between haplotype *h_i_* and *h_j_*. Haplotype network also contains the temporal and spatial distribution of strains in each haplotype, denoted as $p_{te,h_{i}}\left(date\right)$ and $p_{sp,h_{i}}\left(location\right)$, i=0,1,…,m.

More formally, let *S* be a collection of strains with their mutations, locations, and submission dates. The collection of time points was defined as $T=\left\{t_{i}|t=t_{0}+\Delta t\cdot i,i=0,1,\text{…},n-1\right\}$, where Δ*t* represents the time interval, *n* the number of time points, *t_i_* the (*i* + 1)*th* time point. Haplotype networks were constructed at each time point *t_i_* in set *T* (Definition 2).

Definition 2 (Haplotype network at a given time point) Let *S* be a collection of strains with their mutations, locations, and submission dates. The haplotype network of set *S* at a given time point *t* is a haplotype network constructed from strains in set *S* such that their submission dates are no later than *t*.

Specifically, two datasets of haplotype networks were built from the *standard dataset* and the *post‐Omicron dataset*. A series of time points with a 7‐day interval ranging from September 5, 2020, to February 26, 2022, was produced and haplotype networks were constructed from *standard datasets* at those time points (Data S2, Supporting Information). Besides, haplotype networks were constructed from the *post‐Omicron dataset* at time points with a 7‐day interval ranging from November 22, 2021, to July 24, 2023 (Data S4, Supporting Information).

### Feature Extraction

Seven features from the haplotype network, including betweenness, out‐degree, depth, number of sequences, geographic information entropy, the proportion of new sequences, and weighted depth (Supplementary Note 1, Supporting Information) were extracted using igraph,^[^ ^33^ ^]^ which is a collection of network analysis tools. It is important to note that these features were calculated based on the following information without considering the specific mutations in each haplotype and edge:

- The collection of haplotype pairs connected by edges (edges set *E*)
- The node weights and edge weights (*w_h_* and *w_e_*)
- The temporal and spatial distribution of haplotypes ($p_{te,h_{i}}\left(date\right)$ and $p_{sp,h_{i}}\left(location\right)$)

Focusing on the emerging variants within a specific period, features were extracted only from the active haplotypes (Definition 3).

Definition 3 (Active haplotype) Let *G* be a haplotype network, constructed at a given time point *t* from *S*, a collection of strains with their mutations, locations, and submission dates. For a given length of time $\Delta \tilde{t}$, an active haplotype *h* is a haplotype in the haplotype network *G*, such that there exists at least one strain *s* in the haplotype *h*, satisfying $t-\Delta \tilde{t}\leq t_{s}\leq t$, where *t_s_* is the submission date of strain *s*.

### Label Assignment

To simplify the high‐risk variants detection problem, SARS‐CoV‐2 haplotypes were divided into two risk levels: high‐risk and low‐risk. This differs from the four risk levels (VOC, VOI, VUM, and Others) established by WHO. On the *standard dataset*, haplotypes belonging to VOC or VOI were assigned as high‐risk variants, while those belonging to VUM and others were assigned as low‐risk variants (Table S1, Supporting Information, explained in Supplementary Note 3, Supporting Information). However, on the *post‐Omicron dataset*, haplotypes belonging to VOI or VUM were assigned as high‐risk variants, while all other haplotypes were assigned as low‐risk variants (Table S6, Supporting Information). The reason for using different classification criteria for different datasets was that as of July 2023, no SARS‐CoV‐2 variant has been classified as VOC in the new tracking system of WHO.

### HiRisk‐Detector for Detecting High‐Risk Variants of SARS‐CoV‐2

HiRisk‐Detector was developed for detecting high‐risk variants of SARS‐CoV‐2, which leveraged the haplotype network features (Figure 3; Figure S7, Supporting Information).

### HiRisk‐Detector for Detecting High‐Risk Variants of SARS‐CoV‐2—Test HiRisk‐Detector on the Standard Dataset

First, a series of *surveillance time points* were generated with a 7‐day interval ranging from September 5, 2020, to February 26, 2022, to regularly surveil the evolution and risk of SARS‐CoV‐2. Second, the initial model was trained on active haplotypes in each haplotype network constructed at *surveillance time points* from September 5, 2020, to December 19, 2020. December 19, 2020, was the earliest surveillance time point after WHO designed the first VOC/VOI. Third, at each surveillance time point *t_i_* after December 18, 2020, a haplotype network was constructed using all sequences available at *t_i_*, five selected features were extracted from each active haplotype in the constructed haplotype network at *t_i_* and the risk score was predicted for each active haplotype by the trained model. Haplotypes with a risk score higher than 0.5 were defined as high‐risk variants. After that, it was determined whether WHO designed a new VOC/VOI between *t_i_* and *t_i_* − Δt, where Δt equals to seven days. If so, the model was retrained with all active haplotypes in each haplotype network constructed at each surveillance time point no later than *t_i_*. the Python package Statannotations (available at https://pypi.org/project/statannotations/) was utilized to calculate the statistical significance between high‐ and low‐risk haplotypes.

### HiRisk‐Detector for Detecting High‐Risk Variants of SARS‐CoV‐2—Test HiRisk‐Detector on Post‐Omicron Dataset

A series of haplotype networks were constructed from the *post‐Omicron dataset* (Data S3, Supporting Information) at time points with a 7‐day interval ranging from November 22, 2021, to July 24, 2023 (Data S4, Supporting Information). The performance was evaluated on the *post‐Omicron dataset*, and labels were assigned according to WHO's new tracking system (Table S6, Supporting Information). The time points for detecting high‐risk haplotypes range from March 20, 2023, to July 24, 2023, with a 7‐day interval.

## Conflict of Interest

The authors declare no conflict of interest.

## Author Contributions

L.L., C.L., and N.L. contributed equally to this work. Conceptualization, bioinformatics analysis: S.H.S., L.L., C.P.L., and N.L.; wrote original draft: S.H.S. and L.L.; performed Web development: D.Z.; reviewed and edited the original manuscript: Y.B.X., Z.Z., Y.M.B., W.M.Z., and H.L.

## Supporting information



Supporting Information

## Data Availability

Source code of HiRisk‐Detector is written in Python and available at GitHub (https://github.com/Theory‐Lun/HiRiskPredictor), BioCode (https://ngdc.cncb.ac.cn/biocode/tools/BT007386), and Zenodo (https://zenodo.org/records/10060683) under the MIT license. Web server of HiRisk‐Detector is available at https://ngdc.cncb.ac.cn/ncov/monitoring/risk. All complete and high‐quality SARS‐CoV‐2 genome sequences and associated metadata were downloaded from RCoV19 (https://ngdc.cncb.ac.cn/ncov/). The number of daily confirmed cases and deaths of COVID‐19 were downloaded from WHO (https://covid19.who.int/data). The alias of PANGO lineages were downloaded from https://github.com/cov‐lineages/pango‐designation/.

## References

[1] P. Wang , M. S. Nair , L. Liu , S. Iketani , Y. Luo , Y. Guo , M. Wang , J. Yu , B. Zhang , P. D. Kwong , B. S. Graham , J. R. Mascola , J. Y. Chang , M. T. Yin , M. Sobieszczyk , C. A. Kyratsous , L. Shapiro , Z. Sheng , Y. Huang , D. D. Ho , Nature 2021, 593, 130. 33684923 10.1038/s41586-021-03398-2

[2] Eurosurveillance editorial team , Eurosurveillance 2021, 26, 2101211.33478621

[3] M. M. DeGrace , E. Ghedin , M. B. Frieman , F. Krammer , A. Grifoni , A. Alisoltani , G. Alter , R. R. Amara , R. S. Baric , D. H. Barouch , J. D. Bloom , L.‐M. Bloyet , G. Bonenfant , A. C. M. Boon , E. A. Boritz , D. L. Bratt , T. L. Bricker , L. Brown , W. J. Buchser , J. M. Carreño , L. Cohen‐Lavi , T. L. Darling , M. E. Davis‐Gardner , B. L. Dearlove , H. Di , M. Dittmann , N. A. Doria‐Rose , D. C. Douek , C. Drosten , V.‐V. Edara , et al., Nature 2022, 605, 640.35361968

[4] G. Nicora , M. Salemi , S. Marini , R. Bellazzi , BMJ Health Care Inform. 2022, 29, 100643. 10.1136/bmjhci-2022-100643 PMC9742845 36593658

[5] J. Li , Y.‐N. Wu , S. Zhang , X.‐P. Kang , T. Jiang , Brief. Bioinform. 2022, 23, bbac036. 35233612 10.1093/bib/bbac036 PMC9116219

[6] Q. Sun , C. Shu , W. Shi , Y. Luo , G. Fan , J. Nie , Y. Bi , Q. Wang , J. Qi , J. Lu , Y. Zhou , Z. Shen , Z. Meng , X. Zhang , Z. Yu , S. Gao , L. Wu , J. Ma , S. Hu , Nucleic Acids Res. 2022, 50, D888. 34634813 10.1093/nar/gkab921 PMC8728250

[7] M. C. Maher , I. Bartha , S. Weaver , J. di Iulio , E. Ferri , L. Soriaga , F. A. Lempp , B. L. Hie , B. Bryson , B. Berger , D. L. Robertson , G. Snell , D. Corti , H. W. Virgin , S. L. Kosakovsky Pond , A. Telenti , Sci. Transl. Med. 2022, 14, abk3445. 10.1126/scitranslmed.abk3445 PMC8939770 35014856

[8] F. Obermeyer , M. Jankowiak , N. Barkas , S. F. Schaffner , J. D. Pyle , L. Yurkovetskiy , M. Bosso , D. J. Park , M. Babadi , B. L. MacInnis , J. Luban , P. C. Sabeti , J. E. Lemieux , Science 2022, 376, 1327. 35608456 10.1126/science.abm1208 PMC9161372

[9] K. Beguir , M. J. Skwark , Y. Fu , T. Pierrot , N. L. Carranza , A. Laterre , I. Kadri , A. Korched , A. U. Lowegard , B. G. Lui , B. Sänger , Y. Liu , A. Poran , A. Muik , U. Sahin , Comput. Biol. Med. 2023, 155, 106618. 36774893 10.1016/j.compbiomed.2023.106618 PMC9892295

[10] S. Harari , D. Miller , S. Fleishon , D. Burstein , A. Stern , Nat. Commun. 2024, 15, 648. 38245511 10.1038/s41467-024-44803-4 PMC10799923

[11] P. Forster , L. Forster , C. Renfrew , M. Forster , Proc. Natl. Acad. Sci. USA 2020, 117, 9241. 32269081 10.1073/pnas.2004999117 PMC7196762

[12] S. Azad , S. Devi , J. Travel Med. 2020, 27, taaa130. 32776124 10.1093/jtm/taaa130 PMC7454757

[13] S. Song , C. Li , L. Kang , D. Tian , N. Badar , W. Ma , S. Zhao , X. Jiang , C. Wang , Y. Sun , W. Li , M. Lei , S. Li , Q. Qi , A. Ikram , M. Salman , M. Umair , H. Shireen , F. Batool , B. Zhang , H. Chen , Y.‐G. Yang , A. A. Abbasi , M. Li , Y. Xue , Y. Bao , Genomics Proteomics Bioinf. 2021, 19, 727. 10.1016/j.gpb.2021.08.007 PMC8546014 34695600

[14] M. Koutrouli , E. Karatzas , D. Paez‐Espino , G. A. Pavlopoulos , Front. Bioeng. Biotechnol. 2020, 8, 34. 32083072 10.3389/fbioe.2020.00034 PMC7004966

[15] C. Li , L. Ma , D. Zou , R. Zhang , X. Bai , L. Li , G. Wu , T. Huang , W. Zhao , E. Jin , Y. Bao , S. Song , Genomics Proteomics Bioinf. 2023, 21, 1066. 10.1016/j.gpb.2023.10.004 PMC10928372 37898309

[16] T. Kawano‐Sugaya , K. Yatsu , T. Sekizuka , K. Itokawa , M. Hashino , R. Tanaka , M. Kuroda , G3 (Bethesda) 2021, 11, jkab126. 33892501 10.1093/g3journal/jkab126 PMC8135534

[17] L. Van der Maaten , G. Hinton , J. Mach. Learn. Res. 2008, 9, 2579.

[18] F. Pedregosa , J. Mach. Learn. Res. 2011, 12, 2825.

[19] G. Ke , Q. Meng , T. Finley , T. Wang , W. Chen , W. Ma , Q. Ye , T. Y. Liu , Advances in Neural Information Processing Systems, Curran Associates, Inc., Red Hook, New York, 2017.

[20] T. K. Ho , in Proceedings of 3rd international conference on document analysis and recognition , 1995.

[21] D. R. Cox , J. Royal Stat. Soc. 1958, 20, 215.

[22] G. Haixiang , L. Yijing , J. Shang , G. Mingyun , H. Yuanyue , G. Bing , Expert Syst. Appl. 2017, 73, 220.

[23] A. R. Templeton , K. A. Crandall , C. F. Sing , Genetics 1992, 132, 619. 1385266 10.1093/genetics/132.2.619 PMC1205162

[24] H. J. Bandelt , P. Forster , A. Röhl , Mol. Biol. Evol. 1999, 16, 37. 10331250 10.1093/oxfordjournals.molbev.a026036

[25] L. Li , B. Xu , D. Tian , A. Wang , J. Zhu , C. Li , N. Li , W. Zhao , L. Shi , Y. Xue , Z. Zhang , Y. Bao , W. Zhao , S. Song , Brief Bioinform. 2023, 24, bbad174. 37170752 10.1093/bib/bbad174 PMC10199771

[26] T. N. Kipf , M. Welling , arXiv 1609.02907, 2016.

[27] S. Song , L. Ma , D. Zou , D. Tian , C. Li , J. Zhu , M. Chen , A. Wang , Y. Ma , M. Li , X. Teng , Y. Cui , G. Duan , M. Zhang , T. Jin , C. Shi , Z. Du , Y. Zhang , C. Liu , R. Li , J. Zeng , L. Hao , S. Jiang , H. Chen , D. Han , J. Xiao , Z. Zhang , W. Zhao , Y. Xue , Y. Bao , Genomics Proteomics Bioinf. 2020, 18, 749. 10.1016/j.gpb.2020.09.001 PMC7836967 33704069

[28] W. M. Zhao , S. H. Song , M. L. Chen , D. Zou , L. N. Ma , Y. K. Ma , R. J. Li , L. L. Hao , C. P. Li , D. M. Tian , B. X. Tang , Y. Q. Wang , J. W. Zhu , H. X. Chen , Z. Zhang , Y. B. Xue , Y. M. Bao , Yi Chuan 2020, 42, 212. 32102777 10.16288/j.yczz.20-030

[29] Á. O'Toole , E. Scher , A. Underwood , B. Jackson , V. Hill , J. T. McCrone , R. Colquhoun , C. Ruis , K. Abu‐Dahab , B. Taylor , C. Yeats , L. du Plessis , D. Maloney , N. Medd , S. W. Attwood , D. M. Aanensen , E. C. Holmes , O. G. Pybus , A. Rambaut , Virus Evol. 2021, 7, veab064. 34527285 10.1093/ve/veab064 PMC8344591

[30] A. Rambaut , E. C. Holmes , Á. O'Toole , V. Hill , J. T. McCrone , C. Ruis , L. du Plessis , O. G. Pybus , Nat. Microbiol. 2021, 6, 415. 33514928 10.1038/s41564-021-00872-5 PMC7845574

[31] A. Rambaut , E. C. Holmes , Á. O'Toole , V. Hill , J. T. McCrone , C. Ruis , L. du Plessis , O. G. Pybus , Nat. Microbiol. 2020, 5, 1403. 32669681 10.1038/s41564-020-0770-5 PMC7610519

[32] D. H. Huson , R. Rupp , C. Scornavacca , Phylogenetic networks: concepts, algorithms and applications, Cambridge University Press, Cambridge 2010.

[33] G. Csardi , T. Nepusz , InterJournal, Complex Syst. 2006, 1695, 1.

