# Supplementary material for: Machine Learning Early Detection of SARS‐CoV‐2 High‐Risk Variants
Source: Adv Sci (Weinh). 2024 Oct 14;11(45):2405058. doi: 10.1002/advs.202405058 (PMC11615786; doi:10.1002/advs.202405058)
Supplement: Supplementary file 1 — Supporting Information [file ADVS-11-2405058-s001.docx]

**Supplementary Materials for**

**Machine learning early detection of SARS-CoV-2 high-risk variants**

Lun Li^1,2,#^, Cuiping Li^1,2,#^, Na Li^1,2,#^, Dong Zou^1,2^, Wenming Zhao^1,2,3,4^, Hong Luo^1,2^, Yongbiao Xue^1,2,4,*^, Zhang Zhang^1,2,3,4,*^, Yiming Bao^1,2,3,4,*^, Shuhui Song^1,2,3,4,*,+^

^1^ *China National Center for Bioinformation, Beijing 100101, China*

*^2^ National Genomics Data Center, Beijing Institute of Genomics, Chinese Academy of Sciences, Beijing 100101, China*

^3^ *CAS Key Laboratory of Genome Sciences and Information, Beijing Institute of Genomics, Chinese Academy of Sciences, Beijing 100101, China*

^4^ *University of Chinese Academy of Sciences, Beijing 100049, China*

^#^ Equal contribution.

^*^ Corresponding authors.

^+^ Leading contact.

E-mail: [songshh@big.ac.cn](mailto:songshh@big.ac.cn) (Song S), [baoym@big.ac.cn](mailto:baoym@big.ac.cn) (Bao Y), [zhangzhang@big.ac.cn](mailto:zhangzhang@big.ac.cn) (Zhang Z), and [ybxue@big.ac.cn](mailto:ybxue@big.ac.cn) (Xue Y)

The supplementary materials include:

1. Supplementary Notes 1 to 3

2. Supplementary Figures 1 to 7

3. Supplementary Tables 1 to 6

4. Supplementary Data 1 to 4

**Supplementary Note 1: Detailed description of haplotype network features**

Here, we describe the definition of the seven haplotype features, including betweenness, out-degree, depth, number of sequences, geographic information entropy, proportion of new sequences, and weighted depth, in detail.

Formally, let $G=\left( V,E,w_{v},w_{e},p_{te,h_{i}},p_{sp,h_{i}} \right)$ be a haplotype network at time $t$ constructed from a collection of strains $S$, where $V$ is a collection of haplotypes, $E$ is a collection of edges, $w_{v}\left( \cdot\right)$ is node weight, $w_{e}\left( \cdot\right)$ is edge weight, $p_{te,h_{i}}\left( date \right)$ is temporal distribution, and $p_{sp,h_{i}}\left( location \right)$ is spatial distribution (see Definition 1, 2). Note that, in this work, haplotype $G$ is a directed, rooted tree (also called arborescence) since we construct haplotype networks by McAN. The root of $G$ is denoted by $h_{r}$.

**Betweenness**

Betweenness of a haplotype is the number of shortest paths in haplotype network $G$ that pass through the haplotype, given by the expression:

$C_{b}\left( h \right)=\sum_{s\neq h\neq t}\frac{\sigma_{st}\left( h \right)}{\sigma_{st}}$, where $h$ is a haplotype in $G$, $\sigma_{st}$ is the total number of shortest paths from node $s$ to node $t$, $\sigma_{st}\left( h \right)$ is the number of those paths that pass through $h$.

**Out-degree**

Out-degree of a haplotype is the number of outward edges from this haplotype, given by the expression:

$outdegree\left( h \right)=|\left\{ e_{ij}=\left( h_{i},h_{j}|e_{ij}\in E,h_{i}=h \right) \right\}|$, where $h$ is a haplotype in $G$, and $|\cdot|$ is the cardinality of a set.

**Depth**

The depth of a haplotype in a haplotype network is the number of edges from the root to the haplotype. Since haplotype networks are arborescence in this work, there is exactly one directed path from the root to the haplotype. Let $P$ be the path from the root $h_{r}$ to a haplotype $h$. The depth of a haplotype $h$ is defined as $depth\left( h \right)=|E\left( P \right)|$, where $E\left( P \right)$ is a collection of edges that path $P$ has, and $|\cdot|$ is the cardinality of a set.

**Number of sequences**

The number of sequences is the number of sequences in the haplotype, that is the node weight of haplotype $h:w_{v}\left( h \right)$.

**Geographic information entropy**

Geographic information entropy of a haplotype is given by the expression:$gie\left( h \right)=-\sum_{location}p_{sp,h}\left( location \right){log}_{e}p_{sp,h}\left( location \right)$, where $p_{sp,h}\left( location \right)$ is the spatial distribution for haplotype $h$.

**Proportion of new sequences**

The proportion of new sequences for a haplotype is defined as $\sum_{t-\Delta\tilde{t}<date\leq t}p_{te,h}\left( date \right)$, where $p_{te,h}\left( \cdot\right)$ is the temporal distribution, $h$ is the haplotype, $\Delta\tilde{t}$ is a given length of time, and $t$ is the time point constructing haplotype network $G$.

**Weighted depth**

The weighted depth of a haplotype in a haplotype network is the summation of weights of edges from the root to the haplotype: $\sum_{e\in E\left( P \right)}w_{e}\left( e \right)$, where $P$ is the only path from the root $h_{r}$ to the haplotype $h$, $E\left( P \right)$ is a collection of edges that path $P$ has, and $w_{e}\left( \cdot\right)$ is the edge weight.

**Supplementary Note 2: Definition of complete and high-quality SARS-CoV-2 genome sequences**

In this study, a SARS-CoV-2 sequence is defined as ‘complete’ if it is longer than 29,000 bp and covers all protein-coding regions of SARS-CoV-2 (nt 266–29674 of GenBank: MN908947.3); otherwise, it is defined as ‘partial’. Furthermore, we consider a sequence to be of ‘high quality’ if it could pass quality control for both unknown bases (Ns) <= 15 and degenerate bases (Ds, more than one possible base at a particular position and sometimes referred as “mixed bases”) <= 50. Otherwise, it is of ‘low quality’.

**Supplementary Note 3: Explanation of the principle for label assignment**

We have categorized the SARS-CoV-2 variants into two risk levels, high-risk and low-risk, diverging from the four risk levels (VOC, VOI, VUM, and Others) established by the WHO. According to the World Health Organization's official definitions available on their website (https://www.who.int/publications/m/item/historical-working-definitions-and-primary-actions-for-sars-cov-2-variants), we classified Variants of Concern (VOCs) and Variants of Interest (VOIs) as high-risk variants. This classification is based on their complete fulfillment of the following two criteria:

- A1. A SARS-CoV-2 variant with genetic changes that are predicted or known to affect virus characteristics such as transmissibility, disease severity, immune escape, diagnostic or therapeutic escape;
- A2. Identified to cause significant community transmission or multiple COVID-19 clusters, in multiple countries with increasing relative prevalence alongside increasing number of cases over time, or other apparent epidemiological impacts to suggest an emerging risk to global public health.

# Supplementary Figures


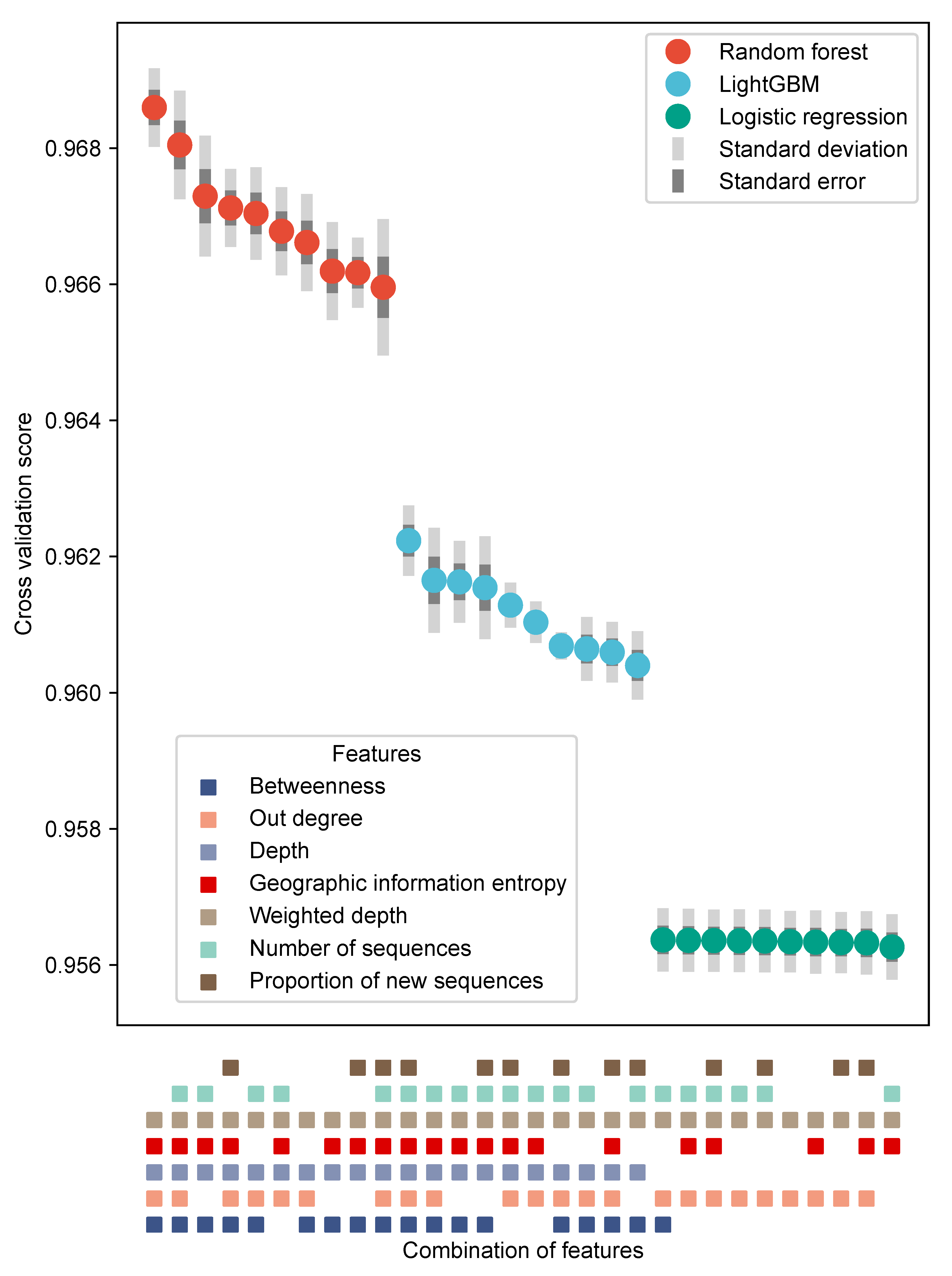


**Figure S1 Cross-validation score from k-fold cross-validation.**

The dots show the average cross-validation score. The light gray and dark gray bars show the standard deviation and standard error, respectively. Only the top 10 combinations of features are shown.


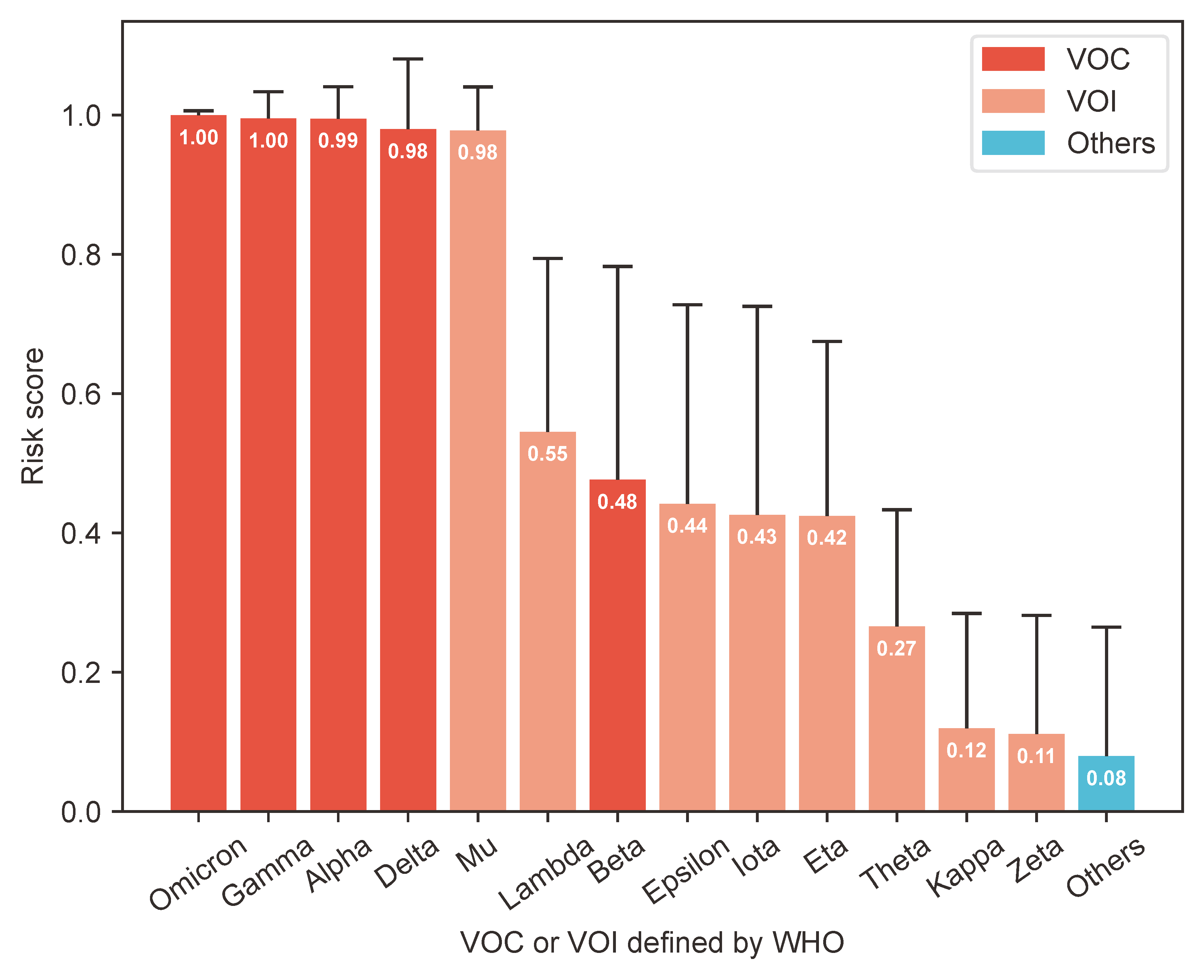


**Figure S2 The average risk score of each VOC/VOI with its standard deviation.**

The height of the bar represents the average risk score of each variant, and the error bar represents its standard deviation. All values are calculated from the experiment of evaluating the effect on data incompleteness and label noise (in section 'Performance with label noise and data incompleteness'). 'Others' represents all variants which is not VOC nor VOI.


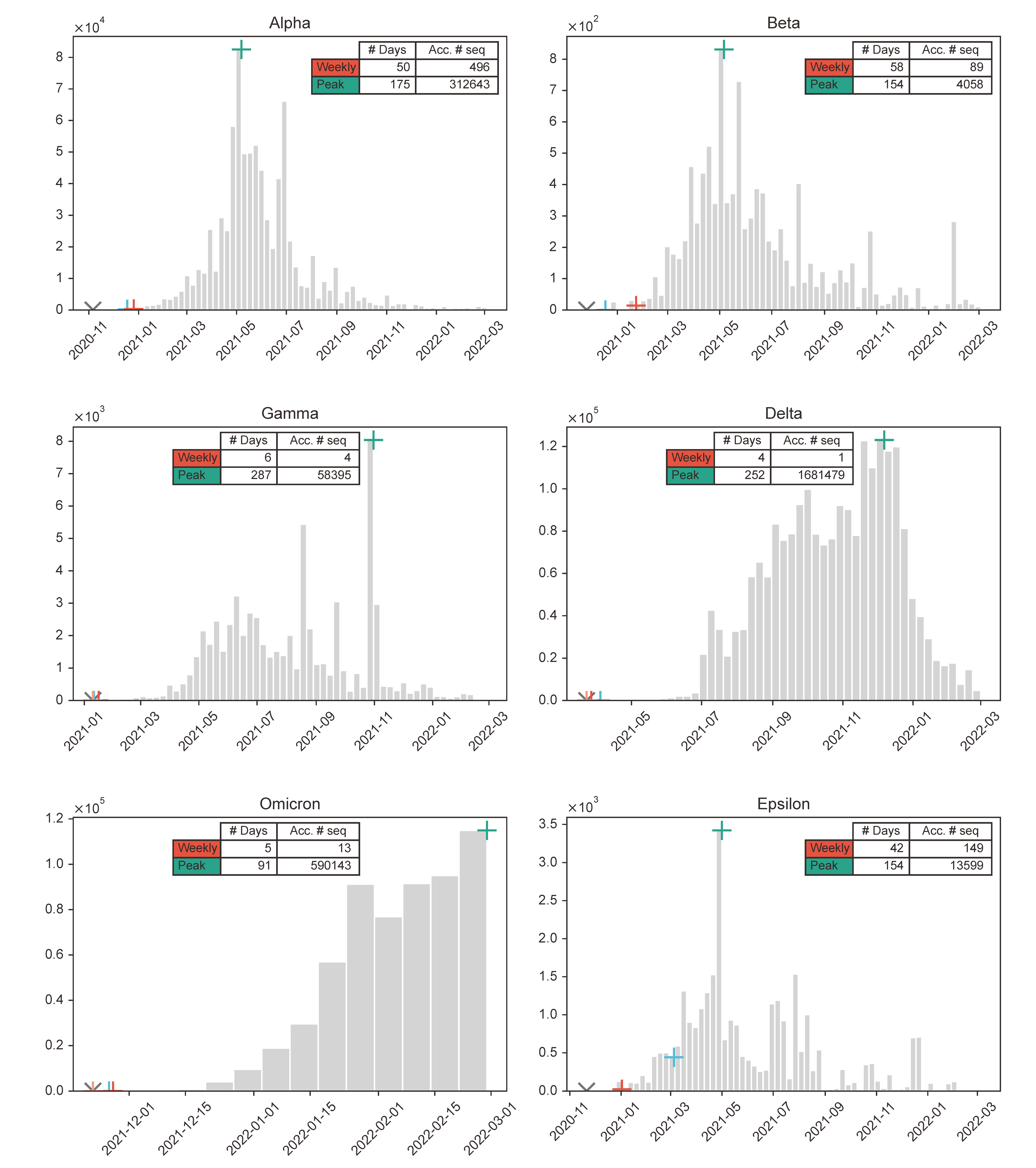


**Figure S3 Number of sequences per week.**

The gray bars show the number of submitted sequences per week. (continued on the next page)


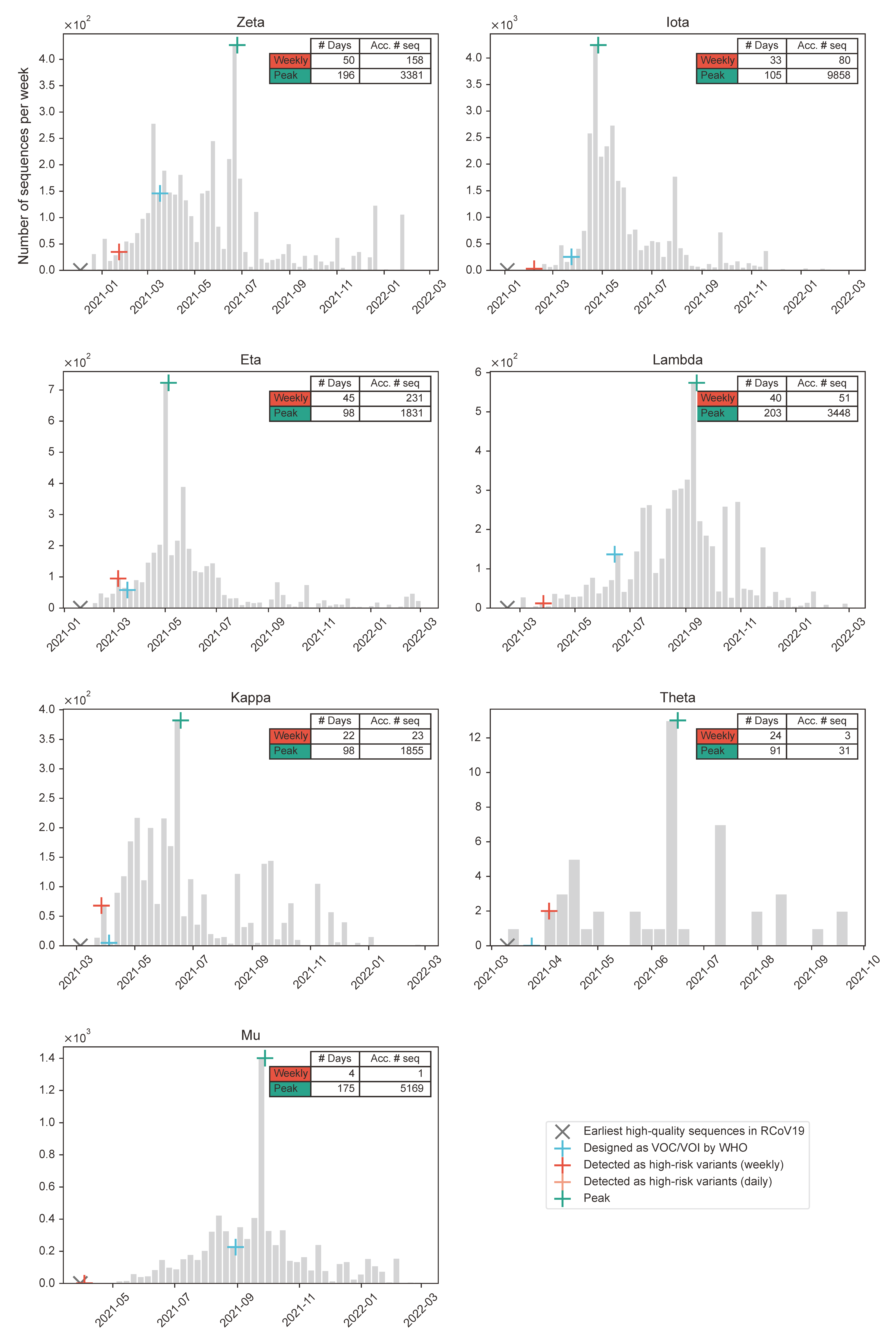


**Figure S3 Number of sequences per week.**

The gray bars show the number of submitted sequences per week. (continued from previous page)


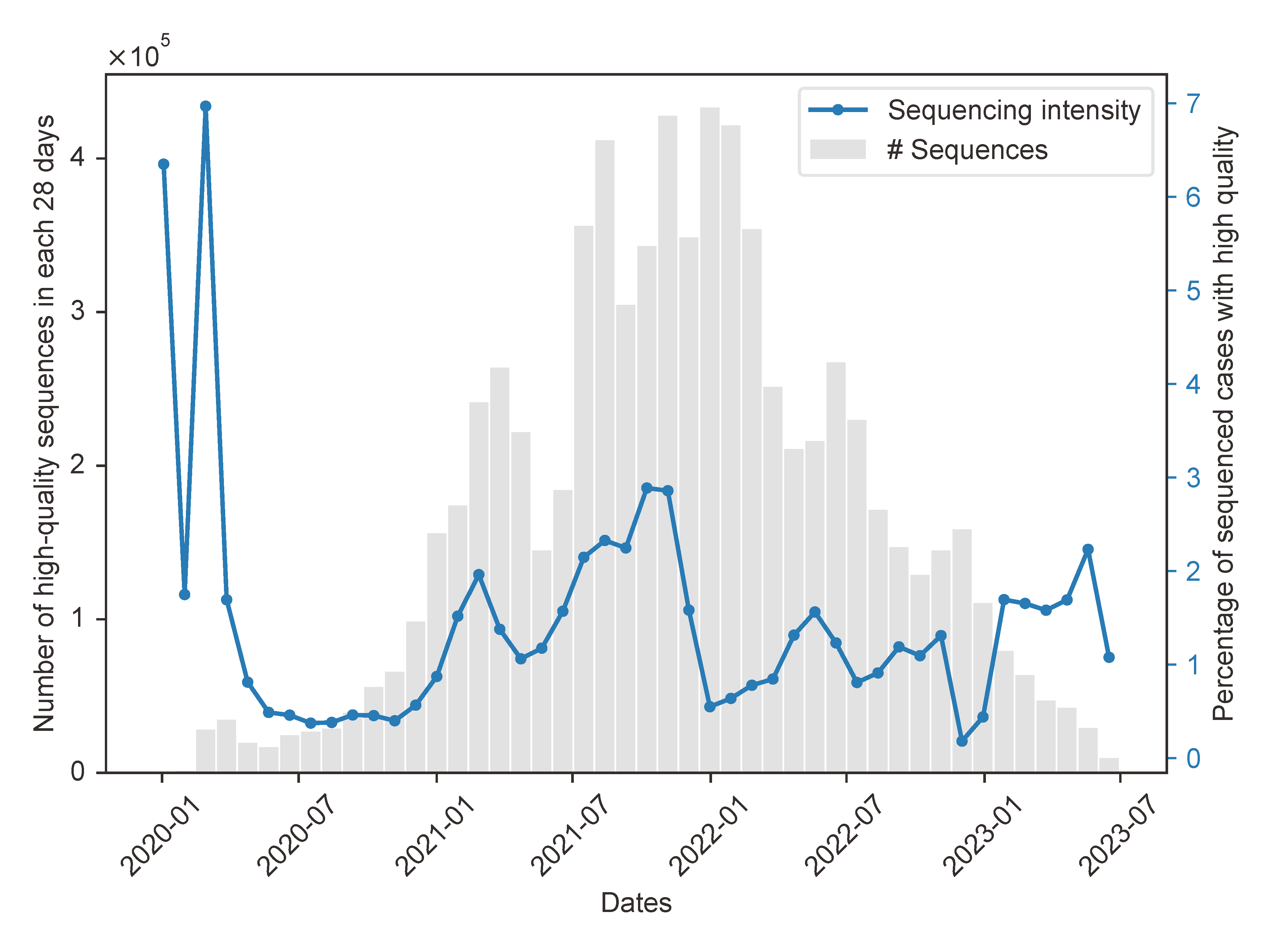


**Figure S4 Sequencing intensity and the number of collected high-quality sequences of SARS-CoV-2 in each 28 days.**

The height of the gray bar represents the number of collected high-quality sequences every 28 days, based on metadata downloaded from RCoV19. The blue dots represent the sequencing intensity of SARS-CoV-2 every 28 days.


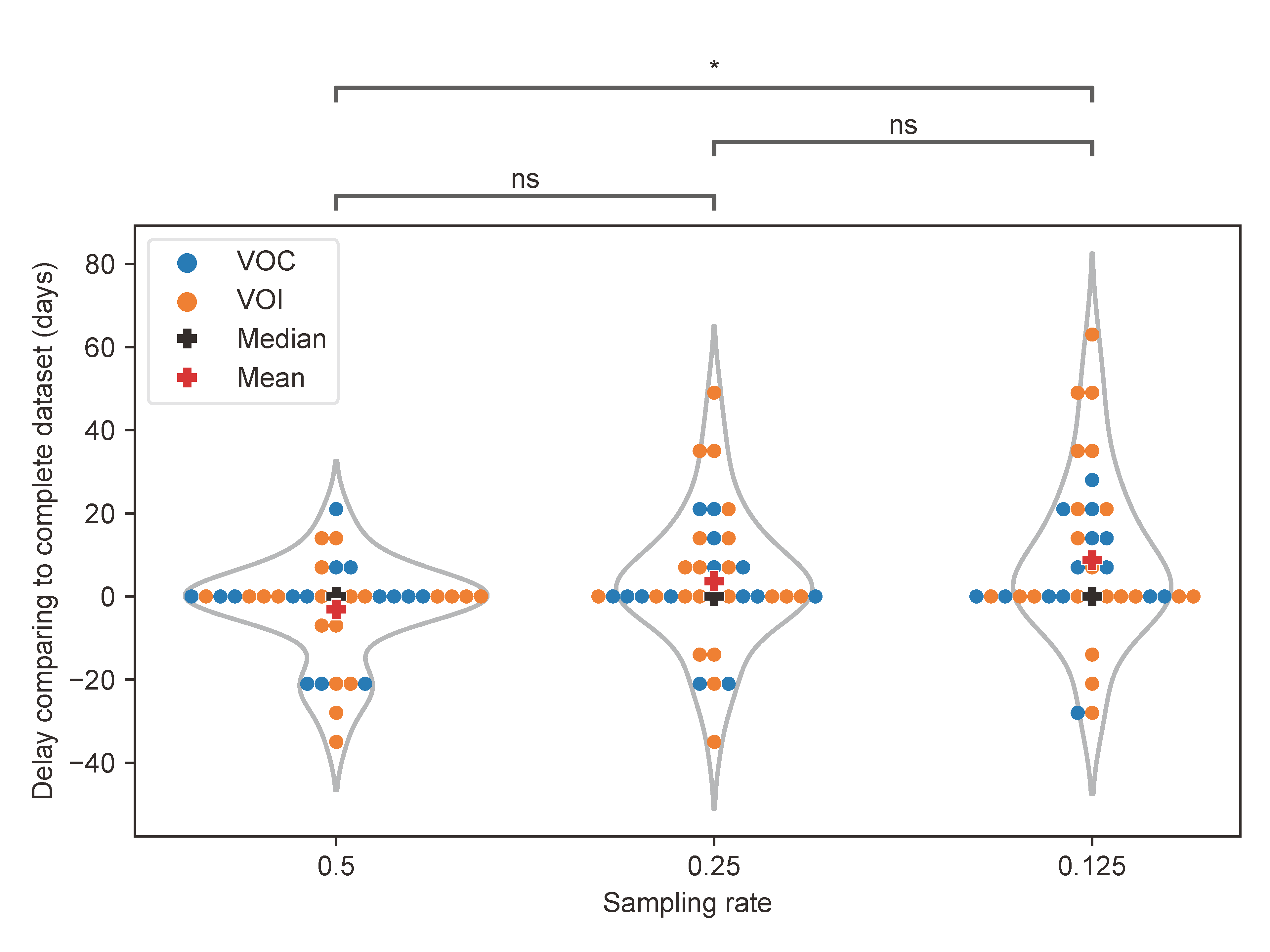


**Figure S5 Delay caused by downsampling.**

The dots show the difference between the earliest time a VOC/VOI was detected as a high-risk variant on the down-sampled standard dataset and the earliest time the VOC/VOI was detected as a high-risk variant on the complete standard dataset. Blue and orange dots represent VOC and VOI, respectively. Black and red crosses represent median and average days of delay, respectively. The *p*-values of the Mann-Whitney test between delays on down-sampled dataset with 50%, 25%, and 12.5% sampling rate are labeled as ns: 5e-2 < p ≤ 1, *: 1e-2 < p ≤ 5e-2, **: 1e-3 < p ≤ 1e-2, ***: 1e-4 < p ≤ 1e-3, ****: p ≤ 1e-4.


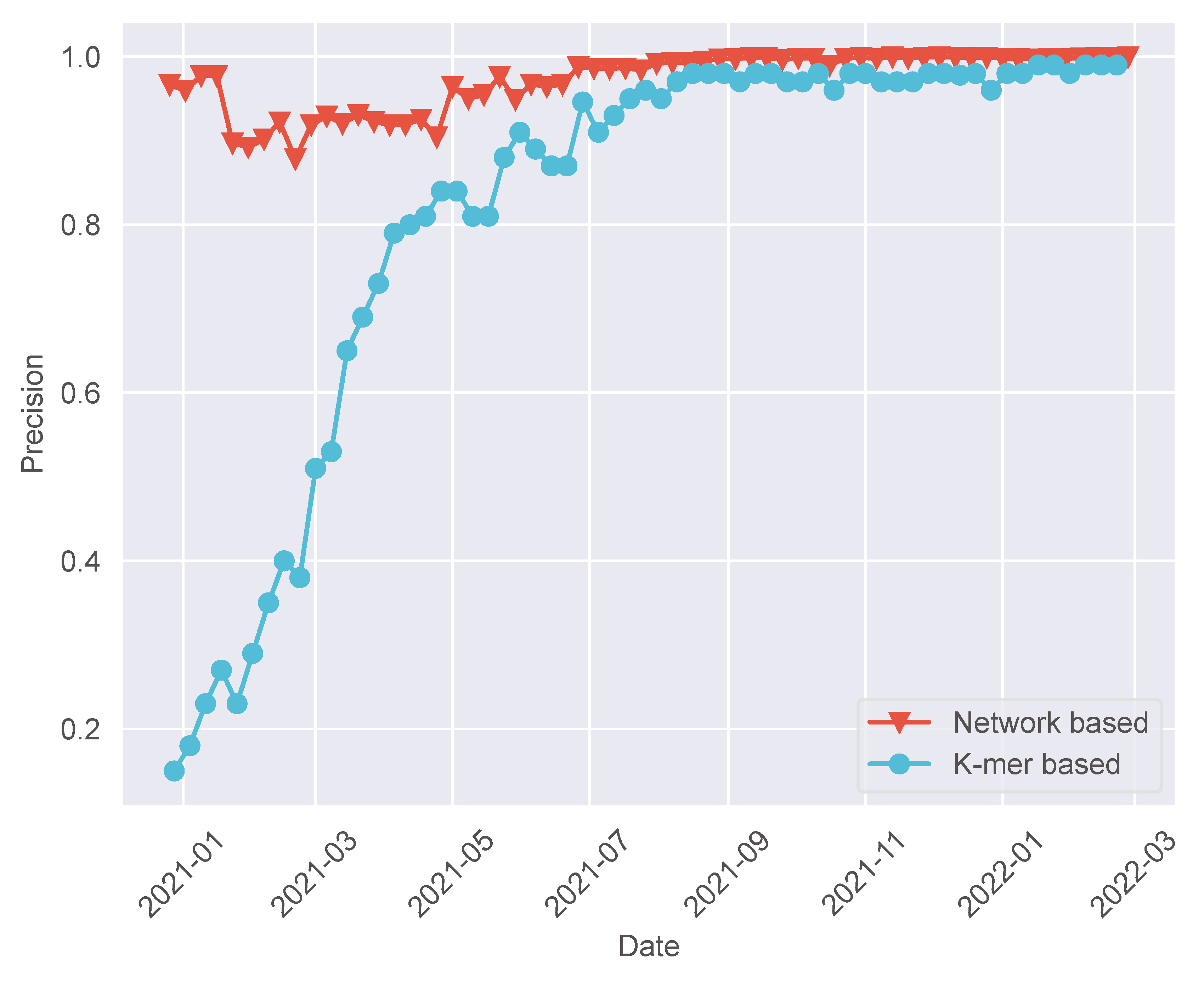


**Figure S6 Comparing the precision of HiRisk-Detector and a k-mer based algorithm on each week.**

The red line indicates the precision of our proposed workflow, and the blue line indicates a detection algorithm based on k-mer.


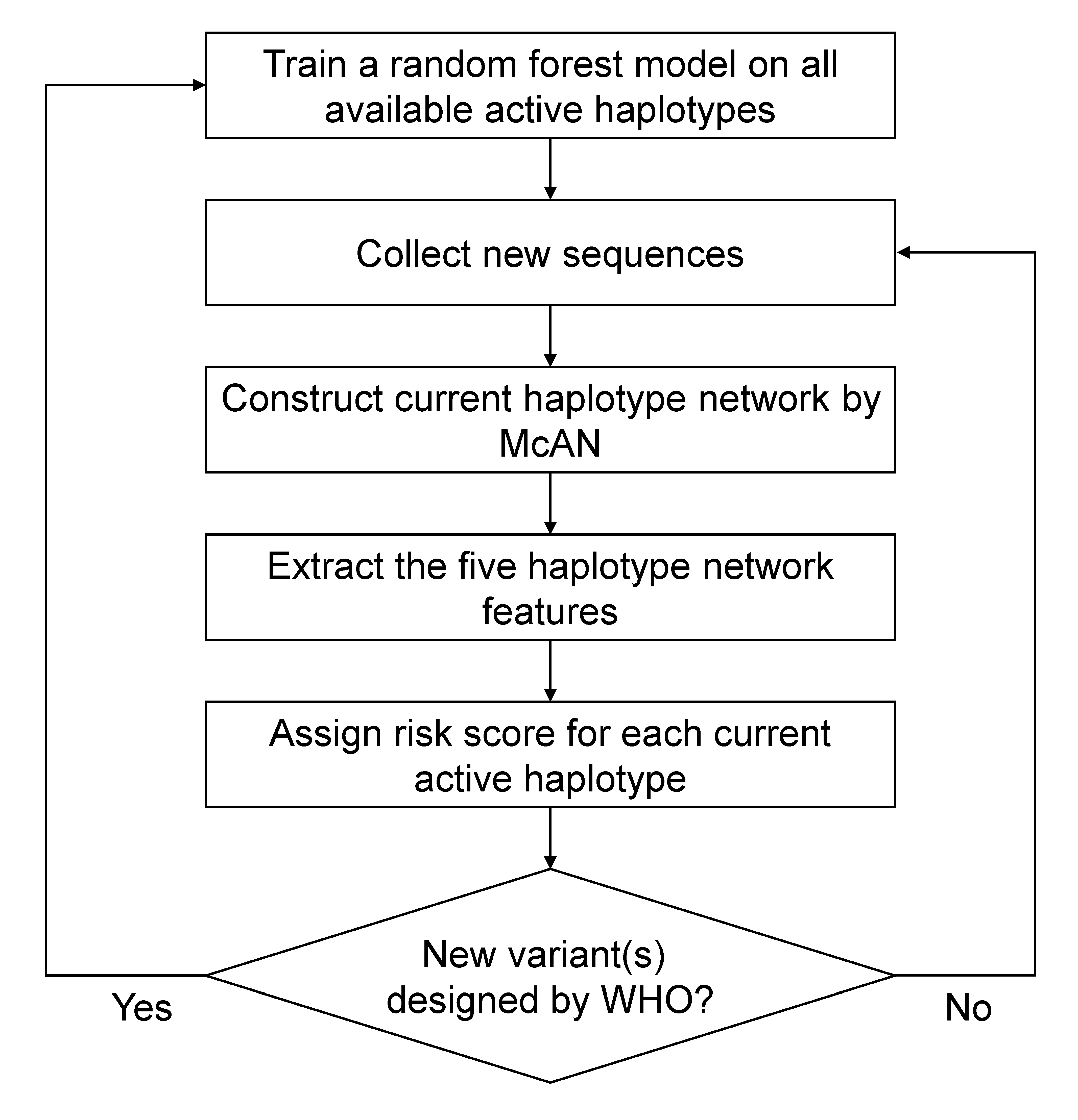


**Figure S7 Illustration of** **HiRisk-Detector.**

# Supplementary Tables

**Table S1 Correspondence among the binary risk level of SARS-CoV-2, WHO risk level, and WHO labels for the standard dataset.** The correspondence between WHO risk and WHO labels is from the WHO’s old tracking system.

| **Binary risk level** | **WHO risk** | **WHO label** |
| --- | --- | --- |
| High risk | VOC | Alpha, Beta, Gamma, Delta, Omicron |
|  | VOI | Epsilon, Zeta, Eta, Theta, Iota, Kappa, Lambda, Mu |
| Low risk | VUM | - |
|  | Others | - |

**Table S2 Information on VOC/VOI designed by WHO.** The 3rd column was calculated based on the submission date extracted from metadata in RCoV19. The date format is year/month/day in this table.

| **WHO label** | **Designed as VOC/VOI by WHO** | **Earliest high-quality strains** | **Delay of WHO (days)** |
| --- | --- | --- | --- |
| Alpha | 2020/12/18 | 2020/11/6 | 42 |
| Beta | 2020/12/18 | 2020/11/26 | 22 |
| Gamma | 2021/1/11 | 2021/1/10 | 1 |
| Delta | 2021/4/4 | 2021/3/23 | 12 |
| Omicron | 2021/11/26 | 2021/11/22 | 4 |
| Epsilon | 2021/3/5 | 2020/11/21 | 104 |
| Zeta | 2021/3/17 | 2020/12/4 | 103 |
| Eta | 2021/3/17 | 2021/1/20 | 56 |
| Iota | 2021/3/24 | 2021/1/4 | 79 |
| Theta | 2021/3/24 | 2021/3/10 | 14 |
| Kappa | 2021/4/4 | 2021/3/5 | 30 |
| Lambda | 2021/6/14 | 2021/2/15 | 119 |
| Mu | 2021/8/30 | 2021/3/30 | 153 |

**Table S3 The performance metrics of HiRisk-Detector on *standard dataset*.**

| **Date** | **PR AUC** | **ROC AUC** | **Precision** |
| --- | --- | --- | --- |
| 2020-12-26 | 0.768 | 0.882 | 0.967 |
| 2021-01-02 | 0.738 | 0.873 | 0.960 |
| 2021-01-09 | 0.803 | 0.900 | 0.977 |
| 2021-01-16 | 0.834 | 0.912 | 0.978 |
| 2021-01-23 | 0.893 | 0.943 | 0.897 |
| 2021-01-30 | 0.892 | 0.944 | 0.892 |
| 2021-02-06 | 0.925 | 0.953 | 0.902 |
| 2021-02-13 | 0.878 | 0.924 | 0.922 |
| 2021-02-20 | 0.874 | 0.921 | 0.878 |
| 2021-02-27 | 0.917 | 0.943 | 0.919 |
| 2021-03-06 | 0.926 | 0.948 | 0.929 |
| 2021-03-13 | 0.955 | 0.965 | 0.920 |
| 2021-03-20 | 0.968 | 0.969 | 0.931 |
| 2021-03-27 | 0.977 | 0.976 | 0.923 |
| 2021-04-03 | 0.989 | 0.987 | 0.919 |
| 2021-04-10 | 0.978 | 0.972 | 0.919 |
| 2021-04-17 | 0.985 | 0.978 | 0.926 |
| 2021-04-24 | 0.965 | 0.963 | 0.904 |
| 2021-05-01 | 0.992 | 0.980 | 0.964 |
| 2021-05-08 | 0.993 | 0.978 | 0.950 |
| 2021-05-15 | 0.990 | 0.966 | 0.955 |
| 2021-05-22 | 0.994 | 0.981 | 0.976 |
| 2021-05-29 | 0.993 | 0.971 | 0.949 |
| 2021-06-05 | 0.996 | 0.979 | 0.967 |
| 2021-06-12 | 0.991 | 0.967 | 0.964 |
| 2021-06-19 | 0.992 | 0.973 | 0.967 |
| 2021-06-26 | 0.997 | 0.988 | 0.988 |
| 2021-07-03 | 0.996 | 0.986 | 0.986 |
| 2021-07-10 | 0.997 | 0.986 | 0.986 |
| 2021-07-17 | 0.995 | 0.977 | 0.986 |
| 2021-07-24 | 0.996 | 0.984 | 0.984 |
| 2021-07-31 | 0.998 | 0.988 | 0.992 |
| 2021-08-07 | 0.999 | 0.993 | 0.993 |
| 2021-08-14 | 0.998 | 0.989 | 0.993 |
| 2021-08-21 | 0.998 | 0.988 | 0.995 |
| 2021-08-28 | 0.998 | 0.975 | 0.997 |
| 2021-09-04 | 0.999 | 0.969 | 0.997 |
| 2021-09-11 | 1.000 | 0.996 | 0.999 |
| 2021-09-18 | 1.000 | 0.993 | 0.999 |
| 2021-09-25 | 0.999 | 0.994 | 0.996 |
| 2021-10-02 | 1.000 | 0.993 | 0.998 |
| 2021-10-09 | 1.000 | 0.997 | 0.998 |
| 2021-10-16 | 1.000 | 0.995 | 0.990 |
| 2021-10-23 | 1.000 | 0.998 | 0.998 |
| 2021-10-30 | 1.000 | 0.998 | 0.999 |
| 2021-11-06 | 1.000 | 0.998 | 0.998 |
| 2021-11-13 | 1.000 | 0.998 | 1.000 |
| 2021-11-20 | 1.000 | 0.996 | 0.998 |
| 2021-11-27 | 1.000 | 0.998 | 0.999 |
| 2021-12-04 | 1.000 | 0.998 | 1.000 |
| 2021-12-11 | 1.000 | 0.998 | 0.999 |
| 2021-12-18 | 1.000 | 0.998 | 0.999 |
| 2021-12-25 | 1.000 | 0.997 | 0.999 |
| 2022-01-01 | 0.999 | 0.953 | 0.998 |
| 2022-01-08 | 0.999 | 0.986 | 0.998 |
| 2022-01-15 | 0.999 | 0.966 | 0.997 |
| 2022-01-22 | 0.999 | 0.986 | 0.998 |
| 2022-01-29 | 0.999 | 0.970 | 0.998 |
| 2022-02-05 | 0.999 | 0.971 | 0.998 |
| 2022-02-12 | 0.999 | 0.923 | 0.999 |
| 2022-02-19 | 1.000 | 0.978 | 0.999 |
| 2022-02-26 | 1.000 | 0.994 | 1.000 |

**Table S4 The mean, median, and mode of delay days caused by the sampling rate on the standard dataset.**

| **Sampling rate** | **Sequencing intensity** | **Mean (days)** | **Median (days)** | **Mode (days)** |
| --- | --- | --- | --- | --- |
| 12.5% | 0.13% | 8.8 | 0 | 0 |
| 25% | 0.25% | 3.7 | 0 | 0 |
| 50% | 0.50% | -3.1 | 0 | 0 |
| 100% | 1.01% | 0 | 0 | 0 |

**Table S5 The performance metrics of HiRisk-Detector on *post-Omicron dataset*.**

| **Date** | **PR AUC** | **ROC AUC** | **F1 score** | **Accuracy** | **Recall** | **Precision** |
| --- | --- | --- | --- | --- | --- | --- |
| 2023-03-20 | 0.989 | 0.991 | 0.968 | 0.966 | 0.995 | 0.942 |
| 2023-03-27 | 0.990 | 0.992 | 0.975 | 0.974 | 0.994 | 0.957 |
| 2023-04-03 | 0.984 | 0.991 | 0.975 | 0.977 | 0.997 | 0.954 |
| 2023-04-10 | 0.993 | 0.995 | 0.978 | 0.978 | 0.991 | 0.965 |
| 2023-04-17 | 0.997 | 0.995 | 0.988 | 0.984 | 0.997 | 0.980 |
| 2023-04-24 | 0.995 | 0.991 | 0.986 | 0.980 | 0.996 | 0.977 |
| 2023-05-01 | 0.995 | 0.995 | 0.987 | 0.984 | 0.998 | 0.976 |
| 2023-05-08 | 0.997 | 0.995 | 0.987 | 0.982 | 0.996 | 0.978 |
| 2023-05-15 | 0.996 | 0.993 | 0.989 | 0.984 | 0.999 | 0.979 |
| 2023-05-22 | 0.996 | 0.990 | 0.990 | 0.984 | 0.998 | 0.982 |
| 2023-05-29 | 0.995 | 0.994 | 0.988 | 0.984 | 0.988 | 0.988 |
| 2023-06-05 | 0.998 | 0.996 | 0.992 | 0.988 | 0.997 | 0.987 |
| 2023-06-12 | 0.999 | 0.997 | 0.995 | 0.991 | 0.998 | 0.992 |
| 2023-06-19 | 0.994 | 0.994 | 0.984 | 0.981 | 0.996 | 0.972 |
| 2023-06-26 | 0.989 | 0.992 | 0.959 | 0.959 | 0.998 | 0.923 |
| 2023-07-03 | 0.995 | 0.992 | 0.990 | 0.986 | 0.999 | 0.982 |
| 2023-07-10 | 0.997 | 0.996 | 0.993 | 0.991 | 0.998 | 0.989 |
| 2023-07-17 | 0.998 | 0.996 | 0.996 | 0.994 | 1.000 | 0.992 |
| 2023-07-24 | 0.995 | 0.987 | 0.987 | 0.979 | 1.000 | 0.975 |

**Table S6 Correspondence among the binary risk level of SARS-CoV-2, WHO risk level, and PANGO lineage for the post-Omicron dataset as of July 2023.** The correspondence between WHO risk and PANGO lineage is from the new tracking system of WHO as of July 2023.

| **Binary risk level** | **WHO risk** | **PANGO lineage** |
| --- | --- | --- |
| High risk | VOC | - |
|  | VOI | XBB.1.5, XBB.1.16 |
|  | VUM | BA.2.75, CH.1.1, XBB, XBB.1.9.1, XBB.1.9.2, XBB.2.3 |
| Low risk | Others | Other PANGO lineages |

# Supplementary Data

**Data S1 (standard dataset)**

The standard dataset includes 4,396,290 high-quality SARS-CoV-2 strains with a submission date no later than February 28, 2022.

It is available at Zenodo (https://zenodo.org/records/10056873, file name: standard_dataset_before_20220228.tsv.tar.gz).

**Data S2 (standard networks dataset)**

A series of haplotype networks constructed from the standard dataset at time points with a 7-day interval ranging from September 5, 2020, to February 26, 2022.

It is available at Zenodo (https://zenodo.org/records/10056873, file name: standard_mcan_features_before_20220228.tar.gz).

**Data S3 (post-Omicron dataset)**

The post-Omicron dataset includes 4,661,821 high-quality SARS-CoV-2 strains submitted between November 22, 2021, and July 27, 2023.

It is available at Zenodo (https://zenodo.org/records/10056873, file name: post_omicron_dataset_after_20211122.tsv.tar.gz).

**Data S4 (post-Omicron network dataset)**

A series of haplotype networks constructed from post-Omicron dataset at time points with a 7-day interval ranging from November 22, 2021, to July 24, 2023.

It is available at Zenodo (https://zenodo.org/records/10056873, file name: post_omicron_mcan_features_after_20211122.tar.gz).
